# Towards Delineating Functions within the *Fasciola* Secreted Cathepsin L Protease Family by Integrating *In Vivo* Based Sub-Proteomics and Phylogenetics

**DOI:** 10.1371/journal.pntd.0000937

**Published:** 2011-01-04

**Authors:** Russell M. Morphew, Hazel A. Wright, E. James LaCourse, Joanne Porter, John Barrett, Debra J. Woods, Peter M. Brophy

**Affiliations:** 1 Institute of Biological, Environmental and Rural Sciences, Aberystwyth University, Aberystwyth, United Kingdom; 2 Liverpool School of Tropical Medicine, Liverpool, United Kingdom; 3 School of Life Sciences, Heriot-Watt University, Edinburgh, United Kingdom; 4 Pfizer Animal Health, Kalamazoo, Michigan, United States of America; McGill University, Canada

## Abstract

**Background:**

*Fasciola hepatica*, along with *Fasciola gigantica*, is the causative agent of fasciolosis, a foodborne zoonotic disease affecting grazing animals and humans worldwide. Pathology is directly related to the release of parasite proteins that facilitate establishment within the host. The dominant components of these excretory-secretory (ES) products are also the most promising vaccine candidates, the cathepsin L (Cat L) protease family.

**Methodology/Principal Findings:**

The sub-proteome of Cat L proteases from adult *F. hepatica* ES products derived from *in vitro* culture and *in vivo* from ovine host bile were compared by 2-DE. The individual Cat L proteases were identified by tandem mass spectrometry with the support of an in-house translated liver fluke EST database. The study reveals plasticity within the CL1 clade of Cat L proteases; highlighted by the identification of a novel isoform and CL1 sub-clade, resulting in a new Cat L phylogenetic analysis including representatives from other adult Cat L phylogenetic clades. Additionally, for the first time, mass spectrometry was shown to be sufficiently sensitive to reveal single amino acid polymorphisms in a resolved 2-DE protein spot derived from pooled population samples.

**Conclusions/Significance:**

We have investigated the sub-proteome at the population level of a vaccine target family using the Cat L proteases from *F. hepatica* as a case study. We have confirmed that *F. hepatica* exhibits more plasticity in the expression of the secreted CL1 clade of Cat L proteases at the protein level than previously realised. We recommend that superfamily based vaccine discovery programmes should screen parasite populations from different host populations and, if required, different host species via sub-proteomic assay in order to confirm the relative expression at the protein level prior to the vaccine development phase.

## Author Summary

Vaccines for neglected parasitic diseases are of paramount importance. An understanding of the basic biology underpinning target expression within parasite populations is one of the pre-requisites for vaccine discovery and development. *Fasciola hepatica* causes global disease in humans and their livestock. The pathology of the disease is associated with the release of cathepsin L (Cat L) proteases from the parasite into the host. The Cat L proteases are the leading vaccine candidates and are split into 5 clades with different functions. The CL1 clade has undergone significant divergence resulting in the formation of sub-clades. We have studied this vaccine candidate family at the population level with proteomic based assays using *F. hepatica* as a case study. We have identified differences in Cat L protein expression profiles between *in vitro* culture compared to *in vivo* host bile, with CL1 members showing greater expression plasticity. Selection pressure exerted by the host driving the divergence of the CL1 clade is revealed by single amino acid polymorphisms. This case study highlights that high resolution population based proteomic assays at the vaccine discovery stage will support the successful development of broad population based commercial vaccines based on defined antigens and their families.

## Introduction

The trematode liver fluke, *Fasciola hepatica*, along with *Fasciola gigantica* are the causative agent of fasciolosis, a foodborne zoonotic disease affecting grazing animals and humans worldwide. The infective metacercariae are ingested by the definitive host where they subsequently excyst in the duodenum. The juvenile fluke migrate to the liver to mature before entering the host bile ducts [1]. Fascioliasis, liver fluke disease, causes annual losses of more than US$3000 million to livestock production worldwide through livestock mortality and by decreased productivity via reduction of milk, wool and meat yields [2]. *F. gigantica* is one of the most important helminth infections of ruminants in Asia and Africa and is most prominent in poorer regions impacting on individual and small farming communities; it inflicts significant losses in cattle, buffaloes, goats and sheep and in India, infection levels can reach 55% in isolated regions [2]. Fasciolosis is a particularly heavy burden in the agricultural based economy of the developing world including India.

*F. hepatica* is also a re-emerging worldwide zoonosis, with estimates of between 2.4 and 17 million people infected worldwide and a further 180 million at risk [3], [4], [5], [6]. Climate changes, altered land use, socio-economic factors and livestock movements provide the opportunity for the increased spread and introduction of pathogenic isolates to humans. The World Health Organisation (WHO) have added fasciolosis to their preventative chemotherapy concept [7] supported by Novartis Pharma AG, with the ultimate aim to implement large scale drug distributions where fasciolosis is a public health concern [8].

Thus, in the absence of commercial vaccines, control of fascioliosis in livestock is based on the use of anthelmintic drugs. The current drug of choice for treatment of fasciolosis is triclabendazole, a benzimidazole-derivative, which shows activity against both juvenile and mature flukes. However, recent reports of triclabendazole resistance have emerged suggesting control of this infection in livestock may become compromised [9], [10], [11], [12]. In addition, consumers worldwide are concerned about drug residues in the environment and food leading to an increased demand for non-chemical based treatments [13]. Research which is directed towards robustly identifying, characterising and validating vaccine candidates is therefore timely.

The pathology associated with fasciolosis is related to the release of proteins from *F. hepatica* directly into the host via specific secretory and non-specific passive processes [14]. The predominant excretory-secretory (ES) products from *in vitro* studies are the cathepsin L (Cat L) proteases [14], [15]. Furthermore, the secreted Cat L proteases from *F. hepatica* appear to have pivotal roles in parasite survival, including immune evasion, nutrition and migration [16]. Additionally, the Cat L proteases are expressed in both juvenile [17], [18] and adult fluke, although the degree of expression, and the isoform expressed, may vary with ontogenic stage [16].

Cat L proteases are found in all liver fluke life stages with many forms expressed and secreted, likely reflecting different biological functions. Unfortunately, singleton sequence deposits in the public domain have caused confusion. *F. hepatica* Cat L public database entries have been deposited with relatively high degrees of sequence similarity between them. For example, according to Tort [19], the Cat L sequences described by Wijffels *et al.* [20] and Roche *et al.* [21] share sequence similarities with Fcp6, described by Heussler and Dobbelaere [22], of greater than 94%. Additionally, an isolated Cat L2 (CL2) clone [21] shows 97% sequence similarity to Fcp1, which was also isolated by Heussler and Dobbelaere [22]. However, phylogenetics can delineate between members of the Cat L protease family, and shows that this is a large family which has expanded within *Fasciola* via gene duplications, leaving a monophyletic group with distinct clade structures [23], [24].

Variable protection rates have been reported using Cat L protease isoforms in vaccine formulations in both field and laboratory trials. A limited understanding of the Cat L protease sub-proteome may be hindering development of this vaccine candidate protein family [25]. A key consideration for vaccine development is to target functional components that are required for the survival of the parasite [26]. To this end, Cat L proteases from *F. hepatica* have been validated as targets. However, an effective broad spectrum commercial vaccine must also overcome the problems of antigenic diversity [27]. Challenge based vaccination trials with a Cat L protease variant derived from a limited liver fluke population analysis may produce variable protection rates as a partial consequence of altered antibody responses [28]. It is clear there will be more Cat L antigen variability within natural liver fluke populations, highlighting that robust vaccine development requires robust population level vaccine discovery with sensitive assay tools.

Therefore, an unbiased global assay of the Cat L proteases, produced *in vivo*, will untangle the complexity of a problematic analysis of individual Cat L proteases in different laboratories. Detailed proteomic experimentation into the Cat L protease family has been performed *in vitro* [24]. Robinson *et al.* [24] identified members of the Cat L protease family from clades 1A (CL1A) and B (CL1B), 2 (CL2) and 5 (CL5), but not from those originating from the newly excysted juvenile (clades 3 and 4) or *F. gigantica* (clades 1C, 3 and 4). However, there are clear discrepancies between ex-host and *in vivo* based studies [14], it is vital to confirm what complement of Cat L proteases are actually expressed in the host environment if vaccines are to be developed on this target. An *in vivo* analysis that avoids the additional non-biologically relevant consequences of ex-host studies will produce more robust datasets for vaccine discovery. Therefore, we incorporate sub-proteomics to delineate the Cat L protease family that are secreted by adult *F. hepatica*, comparing for the first time *in vitro* and *in vivo* Cat L profiles using 2-DE, mass spectrometry, bioinformatics and phylogenetics.

We demonstrate that population level variations in a key parasite vaccine candidate can be revealed by sensitive proteomic level assays. This *Fasciola* case study provides a general strategy to accelerate the pace of vaccine discovery and subsequently vaccine development.

## Materials and Methods

### ES Product Collection and Preparation for 2-DE

Live adult *F. hepatica* were cultured for 4 h and prepared as previously described [14] in order to collect *in vitro* ES products. Gall bladders from naturally infected sheep livers were collected immediately post-slaughter, from a local abattoir, and bile extracted and prepared as previously described in order to obtain *in vivo* ES protein products [14]. Samples prepared for 2-DE SDS-PAGE were re-solubilised in buffer containing 8 M urea, 2% CHAPS w/v, 33 mM DTT, 0.5% carrier ampholytes (pH 3–10, 4.9–5.7 or 5.5–6.7) v/v and protease inhibitors (CompleteMini, Roche, U.K.) for *in vitro* ES products or buffer containing 6 M urea, 1.5 M thiourea, 3% w/v CHAPS, 66 mM DTT, 0.5% v/v carrier ampholytes (pH 3–10, 4.9–5.7 or 5.5–6.7) and protease inhibitors (MiniComplete, Roche, U.K.) for *in vivo* ES products.

### 2-D Electrophoresis

A total of 300 µl of ES product samples were used to actively rehydrate and focus 17 cm linear pH 4–7, 4.9–5.7 or 5.5–6.7 IPG strips (Biorad, U.K.) at 20°C for separation in the first dimension. All IPG strips were focussed between 40,000 and 60,000 Vh using the Ettan IPGphor system (Amersham Biosciences, U.K.). Each IPG strip was equilibrated for 15 minutes in 5 ml of equilibration buffer (containing 50 mM Tris-HCl pH 8.8, 6 M Urea, 30% v/v Glycerol and 2% w/v SDS [29]) with the addition of DTT (Melford, U.K.) at 10 mg/ml. The equilibration buffer containing DTT was removed and replaced with equilibration buffer containing IAA (Sigma, U.K.) at 25 mg/ml again for 15 mins. The IPG strips were separated in the second dimension on the Protean II system (Biorad, U.K.) using 11% polyacrylamide gels and run at 40 mA for approximately 1 h until through the stacking gel followed by 60 mA through the resolving gel until completion.

Gels were Coomassie blue stained (PhastGel Blue R, Amersham Biosciences, U.K.) over night in 10% v/v acetic acid and 30% v/v methanol. The background of coomassie stained gels was removed using 10% v/v acetic acid and 30% v/v methanol leaving visibly stained protein spots. All coomassie stained gels were imaged with a GS-800 calibrated densitometer (Biorad, U.K.) set for coomassie stained gels at 400 dpi. Imaged 2-DE gels were analysed using Progenesis PG220 v.2006. Analysis was performed using the Progenesis ‘Mode of non-spot’ background subtraction method on average gels created from a minimum of three biological replicates. Normalised spot volumes were calculated using the Progenesis ‘Total spot volume multiplied by total area’ method and were used to determine the degree of up and/or down regulation between *in vitro*/*in vivo* comparisons (with significance set at +/−2 fold change). Significance of fold changes was confirmed by a one way ANOVA using LOG_10_ transformation, where appropriate, following a Kolmogorov-Smirnov test for normally distributed spot volumes. Unmatched protein spots were also detected between gel comparisons.

Key protein spots of interest were excised and tryptically digested (Modified trypsin sequencing grade, Roche, U.K.). Briefly, protein spots were destained in 50% v/v acetonitrile and 50% v/v 50 mM ammonium bicarbonate at 37°C until clear. Destained spots were dehydrated in 100% acetonitrile at 37°C for 30 mins followed by rehydration with 50 mM ammonium bicarbonate containing trypsin at 10 ng/µl at 4°C for 45 mins. This was followed by overnight incubation at 37°C. Protein tryptic fragments were then eluted according to Shevchenko *et al.* [30]. Samples were re-suspended in 10 µl of 1% v/v formic acid and 0.5% v/v acetonitrile for tandem mass spectrometry (MSMS).

### Mass Spectrometric Analysis

Samples for MSMS were loaded into gold coated nanovials (Waters, U.K.) and sprayed at 800–900 V at atmospheric pressure using a QToF 1.5 ESI MS (Waters, U.K.). Selected peptides were isolated and fragmented by collision induced dissociation using Argon as the collision gas. Fragmentation spectra were interpreted directly using the Peptide Sequencing programme (MassLynx v 3.5, Waters. U.K.) following spectrum smoothing (2×smooths, Savitzky Golay+/−5 channels), background subtraction (polynomial order 15, 10% below the curve) and processing with Maximum Entropy (MaxEnt) 3 deconvolution software (All MassLynx v 3.5, Waters. U.K.). Sequence interpretation using the Peptide Sequencing programme was conducted automatically with an intensity threshold set at 1 and a fragment ion tolerance set at 0.1 Da. Carbamidomethylation of cysteines, acrylamide modified cysteines and oxidised methionines were taken into account and trypsin specified as the enzyme used to generate peptides. A minimum mass standard deviation was set at 0.025 and the sequence display threshold (% Prob) set at 1. Samples that did not show significant scores and probability when using automated sequence prediction were also interpreted manually to generate sequence tags rather than full peptide sequence information. In these circumstances, the MassLynx program Peptide sequencing was again used with the parameters described above.

### Database Searches and Analysis

Peptide sequences and sequence tags from MSMS were used separately to search the Genbank protein database (www.ncbi.nlm.nih.gov/) using BLAST adjusted for short nearly exact matches [31]. Consequently, all protein accession numbers reported here relate to Genbank. Only peptides with E values of less than 0.1 were used to assign an identity or a clade to a protein (see Table S3 for all E values). In some cases peptides produce E values greater than 0.1 despite 100% sequence matching. As stated, these peptides were not included for Cat L clade assignment but added confidence to the identifications. To identify any novel Cat L isoforms, all sequences that did not show 100% sequence identity to Genbank entries were subjected to a local BLAST analysis using BioEdit Version 7.0.5.3 (10/28/05) [32] searching an in house translated database of *F. hepatica* ESTs (available by anonymous FTP from the Wellcome Trust Sanger Institute ftp://ftp.sanger.ac.uk/pub/pathogens/Fasciola/). Again, only matches with E values less than 0.1 were used to assign a Cat L clade.

### EST Analysis

Peptides sequenced during MSMS analysis in conjunction with the *F. hepatica* EST database yielded a novel Cat L protease sequence. Several of the EST sequences were found to be only partial sequences. Of these matching ESTs the two longest (Fhep22e06 and Fhep21e10) were selected for further sequence confirmation to obtain a more complete sequence. Stratagene BlueScript SK(+) plasmids containing *F. hepatica* inserts (Fhep22e06 and Fhep21e10) were provided by Dr Elizabeth Hoey (Queens University Belfast) were transformed into competent *Escherichia coli* cells (strain DH5α). Fresh plasmid was prepared from 24-hour cultures of transformed single colonies using a Promega (U.K.) Wizard SV Plus MiniPrep DNA purification system according to the manufactures instructions. Forward and reverse DNA sequencing using standard T7 and T3 primers (5′ TAATACGACTCACTATAGGG 3′ T7 primer; 5′ ATTAACCCTCACTAAAGGGA 3′ T3 primer) was performed at the commercial DNA sequencing service of Lark Technologies, Inc. (Essex, U.K.). Nucleotide sequences corresponding to the correct reading frame and similarity were aligned, using BioEdit Version 7.0.5.3 (10/28/05) [32] to establish overlapping regions and facilitate construction of two full-length sequences. For signal peptide prediction the SignalP 3.0 Server [33], available at http://www.cbs.dtu.dk/services/SignalP/, was used. SignalP was set for eukaryotes using both neural networks and hidden Markov models. For epitope prediction, a Kolaskar and Tongaonkar Antigenicity prediction method [34], available at http://tools.immuneepitope.org/tools/bcell/iedb_input, was used.

### Phylogenetics

Alignments of *Fasciola* (*F. hepatica* and *F. gigantica*) Cat L protease nucleotide and amino acid sequences were constructed using ClustalX [35]. The Cat L protease sequences used were taken from the Genbank database (www.ncbi.nlm.nih.gov/) and also included Cat L proteases from this study (EU835857 and EU835858), identified from BLAST analysis searching with novel peptides. Both C-terminal and N-terminal nucleotide sequences were removed where sequence information was limited for many sequences. To construct the nucleotide phylogenetic tree the alignment was exported into Molecular Evolutionary Genetics Analysis (MEGA) software version 4.0 [36]. The phylogenetic tree was generated using a bootstrapped, 1000-replicate, neighbour-joining method. The data were codon based modified using Nei-Gojobori/Jukes-Cantor calculation as a distance based method. Following alignment of amino acid data, amino acid phylogenetic trees were again constructed using MEGA v 4.0. Analysis was performed using a neighbour-joining method, 1000-replicate, bootstrapped tree. The amino acid data was corrected for a gamma distribution (level set at 1.0) and with a Poisson correction.

## Results

### 2-DE Mapping of *F. hepatica* ES

**Figure 1:**
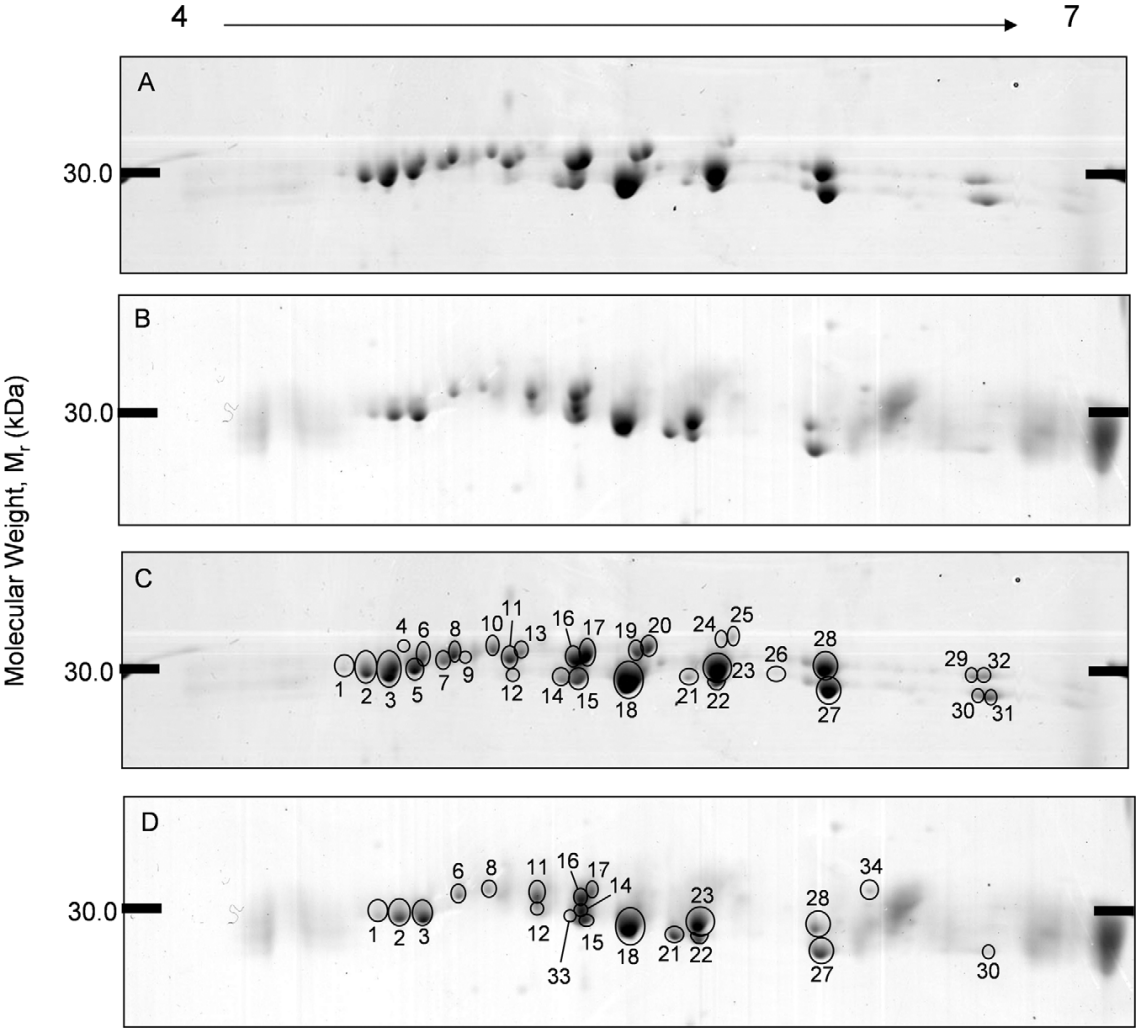
Representative 2-DE protein arrays of *in vitro* and *in vivo* ES Cat L proteases. Proteins were separated across a linear pH range of 4–7 using IEF in the first dimension and 11% SDS-PAGE in the second dimension and Coomassie blue stained. A) & C) 100 µg of *F. hepatica* ES Cat L proteases from *in vitro* culture. B) & D) 250 µg of *Fasciola* ES Cat L proteases from *in vivo* host bile analysis. In both C and D numbered and circled protein spots correspond to putative identifications located in Table 1.

**Figure 2:**
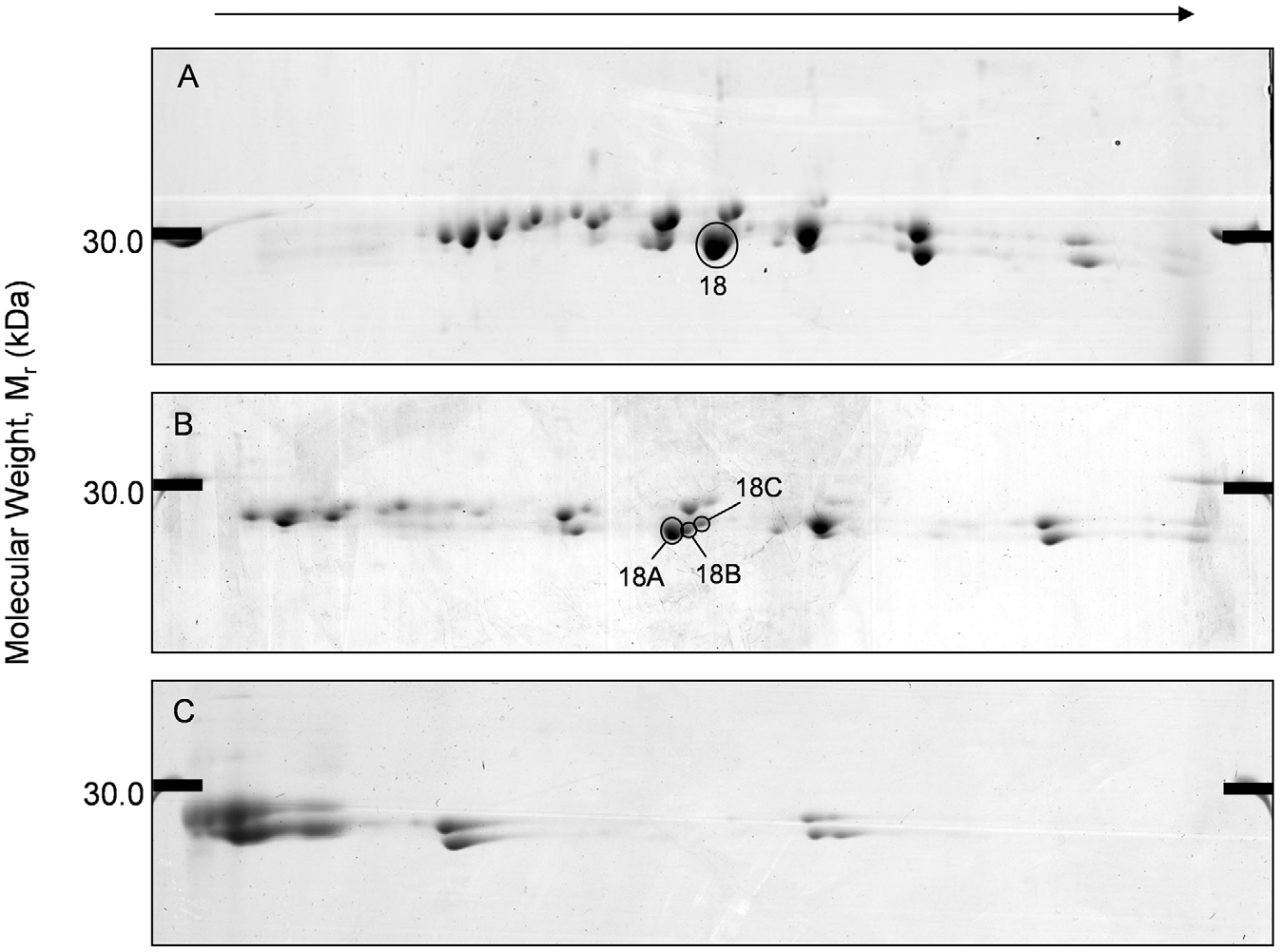
Further resolving the Cat L protease sub-proteome using micro range IPG strips. Representative micro range 2-DE protein arrays of *in vitro* ES Cat L proteases. All 2-DE maps were run with 11% SDS-PAGE in the second dimension and (A) pH 4–7 (B) pH 4.9–5.7 (C) pH 5.5–6.7 in the first dimension. Numbered and circled protein spots correspond to putative identifications located in Table 1.

The *F. hepatica* ES proteins prepared from *in vitro* culture and *in vivo* from host bile were analysed by 2D electrophoresis using methods well developed in our lab. ES protein arrays produced from *in vitro* ES products were highly reproducible, with the average percentage matching between replicates at 91%, and good matching for *in vivo* bile analysis at 75.7%. These 2D arrays of the ES protein revealed a group of protein spots migrating to just below the 30 kDa protein marker and ranging in p*I* from 4.6–6.6 (*in vitro*: p*I* 4.60–6.62. *in vivo*: p*I* 4.65–6.52). This group consisted of 32 protein spots from *in vitro* samples and 20 protein spots from *in vivo* samples (Figure 1). ES samples were also analysed using micro range IPG strips in order to check for potential overlapping or co-migrating protein spots. When using a micro range from pH 4.9 to 5.7, spot 18, previously resolved as one spot (Figure 2A), migrated to produce three distinct spots (Figure 2B). When using IPG strips ranging from pH 5.5 to 6.7, no overlapping protein spots were seen (Figure 2C). All of the fore mentioned protein spots were excised for MSMS analysis to identify the ES Cat L proteases, in total 34 from *in vitro* samples and 22 from *in vivo* samples.

Following MSMS analysis, 30 protein spots from *in vitro* samples and 19 from *in vivo* samples were identified as *F. hepatica* Cat L proteases (Table 1). To assign a Cat L protease to a clade, according to the classification of Robinson *et al.* [24], clade specific peptides were identified (Clade 1: VTGYYTVHSGSEVELK and NSWGLSWGER; Clade 2: VTGYYTVHSGDEIELK, LTHAVLAVGYGSQDGTDYWIVK and HNGLETESYYPYQAVEGPCQYDGR; Clade 5: PDRIDWR and FGLETESSYPYR together with TSISFSEQQLVDCSR). From identifying clade specific peptides, 14 different isoforms of *F. hepatica* Cat L protease isoforms were identified representing the adult CL1, CL2 and CL5 Cat L protease clades. No matches were made to the juvenile clades CL3 and CL4 or from the *F. gigantica* CL1C sub-clade.

**Table 1:** Identification of Cat L proteases from ES preparations by MSMS.

				ES Preparationd)		
Spot Identifier	MS/MS Derived Peptidesa) ^,^ b)	Putitative Identity	Genbank Accession Numberc)	*In Vitro*	*In Vivo*	Clade
1	-	Not Identified	Not Identified	−	+	N/A
2	1- NSWGTWWGEAYTIR	Cathepsin L-like	CAA80445/6	• (5.20)	• (6.29)	CL2
	2- GNMCGIASLASVPMVAR					
	3- YVGNYGCGGGYFEDAYEYLK					
	4- VLAVGYGSQDGTDYWIVK *					
	5- TESYYPYQAVEGP *					
3	1- QDGSGIASLASVPMVAR	Secreted Cathepsin L2	ACC47721	• (9.24)	• (8.78)	CL2
	2- ASASFDTQQLVDCTR					
	3- YVGNYGCGGGYMENAYEYLK					
	4- LTHAVLAVGYGSQDGTDYWIVK					
	5- NQGQCGSCADAFSTTGAVEGQFR					
	6- LGKDHTESYYPYQAVEGPCQYDGR					
4	-	Not Identified	Not Identified	•		N/A
5	1- ASASFSEQQLVD *	Cathepsin L-like	CAA80445/6	• (4.70)		CL2
	2- YMENAYEYLK *					
	3- QAVEGPCQYDGR *					
6	1- FGLETESSYPYR	Cathepsin L-like/Cathepsin L	CAA80447 or AAF76330	• (2.25)	• (3.77)	CL5
	2- FSEQQLVDCSR *					
7	1- ASASFSEQQLVDCTR *	Cathepsin L-like	CAA80445/6	• (1.46)		CL2
	2- GNMCGIASLASVQSGAAR					
	3- VTGYYTVHSGDEIELK *					
	4- GLQDHTESYYPYQAVEGQEQYDGR					
	5- PYQAVEGPCQYDGR *					
8	1- FGLETESSYPYR	Cathepsin L-like/Cathepsin L	CAA80447 or AAF76330	• (2.36)	• (1.84)	CL5
	2- TSISFSEQQLVDCSR					
	3- TSISFSEQQLVGSMSR					
	4- DAPAFMASLASVPMVAQFP					
9	-	Not Identified	Not Identified	•		N/A
10	1- FGLETESSYPYR	Cathepsin L-like/Cathepsin L	CAA80447 or AAF76330	• (0.91)		CL5
	2- TSISFSEQQLVDCSR *					
	1- YPYTAVEGQCR *	Cathepsin L/Cathepsin L-like	NFD			CL1
11	1- NSWGSYWGER	Cathepsin L and Cathepsin L/Cathepsin L-like	NFD	• (2.87)	• (3.61)	CL1A
	2- GYYTVHSGSEVELK *					
	3- TGYYTVHSGSEVELK *					
	4- VTGYYTVHSATTVELK					
	5- IASLASLPMVAR *					
	6- QFGLETESSYPYTAVEGEGEE					
	7- YPYTAVEGQCR *					
12	1- YPYTAVEGQCR *	Cathepsin L (Numerous Types)	NFD	− (0.32)	+ (1.00)	CL1
13	1- QFGLETESSYPY**R** *	Cathepsin L (Numerous Types)	NFD	• (0.23)		CL1
14	1- QFGLETESSYP *	Cathepsin L (Numerous Types)	NFD	• (1.55)	• (4.07)	CL1
15	1- GNFCGIASLASLPFVAR	Cathepsin L	AAR99518	• (3.03)	• (5.25)	CL1A
	2- QFGLETESSYPYTAVEGQGCR					
	3- ARVGSEGPAAVAVDVESPGCYNGAR					
16	1- VTGYYTVHSGSEVELK	Cathepsin L	AAM11647	• (6.61)	• (6.46)	CL1A
	2- GNFCGIASLASLPFVAR					
	3- QFGLETESSYPYTAVEGQCR					
	4- GSCWAFSTTG *					
	5- NSWGSYWGER					
17	1- QFGLETESSYPY**R**	Cathepsin L/Cathepsin L-like/Cathepsin L1	NFD	• (1.85)	• (2.18)	CL1A/B
	2- VTGYYTVHSGSEVELK					
	3- GNFCGIASLASLPFVAR					
	4- ETESSYPYTAVEGQCR *					
18A	1- NSWGLSWGER	Cathepsin L1	CAC12806	• (9.21)	• (14.81)	CL1B
	2- VTGYYTVHSGSE**A**ELK					
	3- VTGYYTVHSGSEVELK					
	4- LSAPWCIASLASLPMVAR					
	5- GNCGSCWAFSTTGTMEGQYMKNEK *					
18B	1- VTGYYTVHSGSVELK	Cathepsin L	AAR99518 OR AAA29136	• (3.23)	• (5.21)	CL1A
	2- QFGLETESSYPYTAVEGQCR					
	3- GPAAVAVDVESDF *					
	4- NCGSCWAFSTTGTMEGQYMKNER *					
18C	1- GNMCGIASLASLSAQGAR	Cathepsin L (Numerous types)	NFD	• (1.57)	• (2.17)	CL1A/B
	2- ETESSYPYTAVEGQCR *					
	3- NCGSCWAFSTTGTMEGQYMKNER *					
19	1- NSWGLSWGER	Cathepsin L	AAM11647	• (4.20)		CL1A
	2- NSWGSYWGER					
	3- TGYYTVHSGSEVELK *					
	4- VTGYYTVHSTATVELK					
	5- GNMCGIASLASLPMVAR					
	6- QFGLETESSYPYTAVLCAQTN					
20	1- QFGLETESSYPY**R**	Cathepsin L/Cathepsin L-like/Cathepsin L1	NFD	• (1.27)		CL1A/B
	2- TGYYTVHSGSEVEL *					
	3- GNMCGIASLASLKTGGAR					
	4- GNRTFTQSSYPYTAVEGTELT					
21	1- FGLETESSYPYT *	Cathepsin L (Numerous Types)	NFD	− (1.24)	+ (3.46)	CL1A/1B
22	1- GYYTLHSGNEAGLK	Cathepsin L-like	ACJ12893/4	• (1.72)	• (2.55)	CL1D
	2- QFGLETESSYPY**R** *					
	3- TGYYTLHSGNEAGLK					
	4- VTGYYTLHSGNEAGLK *					
	5- IASLASLPMVAR *					
	6- GNMCGIASLASL *					
23	1- NSWGSYWGER	Cathepsin L/Cysteine Protease	AAM11647, AAR99518 or AAB30089	• (13.85)	• (12.51)	CL1A
	2- MCGIASLASLPMVAR *					
	3- ETESSYPYTAVEGQCR *					
	4- QFGLETESSYGYSQPEGQCR					
	5- GYGTQGGTDYWIVK *					
24	1- QFGLETESSYPY**R** *	Cathepsin L (Numerous types)	NFD	• (0.47)		CL1
	2- GFCNGIASLASLPMVAR					
25	1- QFGLETESSYPY**R** *	Cathepsin L (Numerous types)	NFD	• (0.28)		CL1
26	-	Not Identified	Not Identified	•		N/A
27	1- GYYTLHSGNEAGLK	Cathepsin L-like	ACJ12893/4	• (6.35)	• (6.01)	CL1D
	2- QFGLEGVGLQLPY**R**					
	3- VTGYYTLHSDANAGLK					
	4- NMCGIASLASLPMVAR *					
	5- GDKSGIASLASLPFVAR					
28	1- SGIYQSQTCSPLR	Cathepsin (Precursor)	AAA29136	+ (8.08)	− (2.94)	CL1A
	2- GNESGIASLASLPFVAR					
	3- QFGLETESSYPYTAVEGGASTQ					
29	1- VTGYYTVHSGSEVELK	Cathepsin L/Cathepsin L-like	NFD	• (1.14)		CL1A/B
30	1- GYYTLHSGNEAGLK	Cathepsin L-like	ACJ12893/4	+ (1.32)	− (0.55)	CL1D
	2- QFGLETESSYPY**R**					
	3- TGYYTLHSGNEAGLK					
	4- VTGYYTLHSGNEAGLK					
	5- GNMCGIASLASLPMVAR					
31	1- GYYTLHSGNEAGLK	Cathepsin L-like	ACJ12893/4	• (0.95)		CL1D
	2- QFGLETESSYPY**R**					
	3- TGYYTLHSGNEAGLK					
	4- VTGYYTLHSGNEAGLK					
	5- VTGYYTLHSGNEAGLK					
32	1- SGIYQSQTCSPLR	Secreted Cathepsin L1/Cathepsin L/Cathepsin	AAB41670, AAP49831, AAM11647, AAA29136	• (0.34)		CL1A
33	-	Not Identified	Not Identified		•	N/A
34	-	Not Identified	Not Identified		•	N/A

Peptide sequences were used to search against Genbank or a translated EST library for the identification of specific Cat L proteases. Single amino acids in bold type in MSMS sequences indicate proteomic identification of single amino acid polymorphisms (SAAP) deviating from published sequences and revealed though a translated EST database. Spots with accession numbers as NFD relate to spots where too few peptides were sequenced preventing isoform and sub-clade identification and were consequently not fully designated (NFD), although the clade could be defined. All data for protein identification, such as percentage coverage and search scores, can be seen in Tables S2 and S3.

a) Sequences derived from MSMS analysis were interpreted either, automated or manually (where manually interpreted using Masslynx version 3.5 sequences are denoted by a *). Sequenced amino acids that match exactly with those found in the Genbank database or translated EST database are underlined.

b) For MSMS spectra from peptides specific to each Cat L isoform see Figures S6, S7, S8, S9, S10, S11.

c) Protein accession numbers correspond to those from Genbank.

d) If protein spots were identified *in vitro* or *in vivo* they are denoted by •, if they are up or down regulated when compared with the other they are denoted with + or − respectively. The percentage contribution of each identified Cat L protease spot compared to the total Cat L proteases calculated using densitometry are in parentheses below the appropriate symbol (•,+ or −).

Despite classifying all the identified Cat L proteases to a Cat L protease clade, the specific isoform or sub-clade could not be assigned, suggesting the specific number of isoforms identified may be under represented. Where possible, MSMS was used to provide sequence information for at least three peptides per spot to definitively identify Cat L proteases. However, in our hands, seven identifications based on single peptides were still able to place a Cat L protease to a single Clade (CL1, CL2 or CL5) although not to a single sub-clade such as CL1A or CL1B. These Cat L protease isoforms were often labelled as ‘NFD’ (not fully designated) to a specific Cat L protease clade. The inability to identify a sub-clade was highlighted from members of the CL1A and 1B clades.

Only two Cat L protease isoforms were identified as single protein spots in the *in vitro* ES and *in vivo* bile 2DE arrays. These were identified as a CL1B isoform (Accession number CAC12806) and a CL2 isoform (Accession number AAC47721). Both of these enzymes account for a vast proportion of the secreted Cat L proteases (CAC12806: 9–15%; AAC47721: 8–9%).

**Figure 3:**
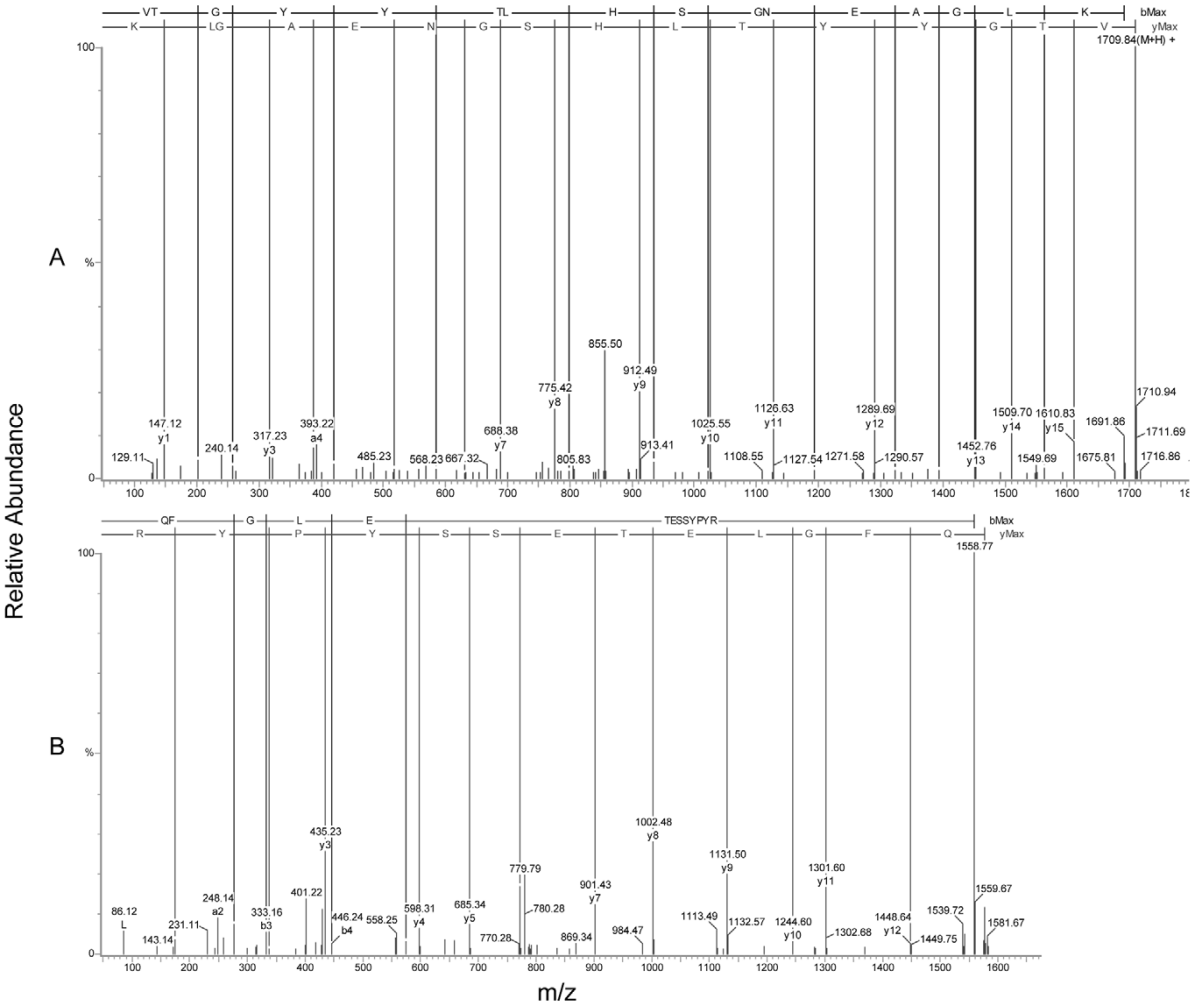
MSMS evidence of novel sequence and SAAP. MSMS spectra from (A) the analysis of a peptide (VTGYYTLHSGNEAGLK) sequenced from spots 22, 27, 30 and 31, belonging to a novel CL1D isoform (B) the analysis of a SAAP variant peptide (QFGLETESSYPYR) sequenced from many protein spots including the novel CL1D isoform. Sequencing was performed both automated and manually (in these cases automated) using MassLynx v 3.5.

Several peptides from spots 22, 27, 30 (both *in vitro* and *in vivo*) and 31 (*in vitro* only) did not appear in any entries in the public domain. Consequently, these peptides were used to locally BLAST a *F. hepatica* EST database (ftp://ftp.sanger.ac.uk/pub/pathogens/Fasciola/). As a result from local searches, identical matches were made with twenty three EST sequences, prior to EST assembly. One specific sequence identified, corresponded to a novel amino acid chain; VTGYYTLHSGNEAGLK (Figure 3A) and led to the identification of a new Cat L protease isoform representing a resembling a CL1 clade member (See section 3.4).

### ES Product Comparison; *In Vitro* versus *In Vivo*

Progenesis PG220 v.2006 gel analysis software was used to identify Cat L proteases with altered expression levels from *in vitro* culture and from *in vivo* samples and Cat L proteases absent or present in either sample. The two samples, *in vivo* ES Cat L proteases (twenty spots) and *in vitro* ES Cat L proteases (thirty two spots), were matched to one another using Progenesis PG220 v. 2006 to give a percentage matching between both of 51.4% matching comparing *in vitro* to *in vivo* and 90% comparing *in vivo* to *in vitro*.

Having matched *in vitro* and *in vivo* preparations to one another, an assessment of the relative quantification of the Cat L proteases could be made. This analysis was conducted using ‘normalised’ spot volumes, which facilitates a relative quantitative assessment despite different protein quantities loaded onto each array (100 µg of *in vitro* ES Cat L proteases versus 250 µg of *in vivo* ES Cat L proteases). From this analysis, five Cat L proteases from *in vivo* preparations show altered expression levels when compared to *in vitro* Cat L proteases (Table 1). Three of which show an increase in relative expression and the remaining two showing decreases (reversed for *in vitro* compared to *in vivo* Cat L proteases). However, following ANOVA with LOG_10_ transformed data, only one change was confirmed as significant; Spot 12 increased *in vivo* (F_1,5_ = 25.90 P = 0.015*) and identified as a CL1 protease. An additional spot approached significance; Spot 1 increased *in vivo* (F_1,5_ = 5.77 P = 0.074) but remaining unidentified. The remaining protein fold changes, Spots 21, 28 and 30, all identified as CL1 members, proved not significant due to a high variance exhibited between replicates. To fully confirm these differential levels of expression (>2 fold) statistically, further sampling would be required.

Four Cat L protease spots were clearly identified *in vitro* and not *in vivo*. These protein spots, 5, 7, 19 and 20, were highly abundant in the *in vitro* samples and absent in bile preparations. All four of these protein spots (5, 7, 19 and 20) had also been identified elsewhere in the ES Cat L profile (in both *in vitro* and *in vivo* samples), spots 5 and 7 (a CL2 member) were previously identified in spot 2, spot 19 (a CL1A member) was previously identified in spot 16, and spot 20, a CL1A/1B member potentially identified in spot 17. A further 7 protein spots identified only *in vitro* samples (10, 13, 24, 25, 29, 31 and 32) were of lower abundance and so cannot been completely regarded as absent from *in vivo* samples. In all cases these 7 proteins were identified as CL1 members with spot 10 also containing a CL5 member.

### Identification of Single Nucleotide Polymorphisms

**Figure 4:**
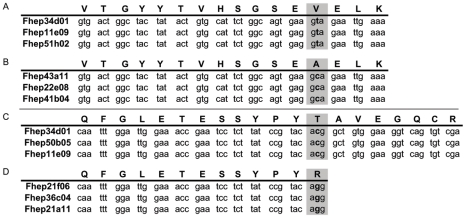
Evidence for SAAPs and non-synonymous single nucleotide polymorphisms (nsSNPs). Amino acid residues and their corresponding nucleotide codons outlined in grey locate the nsSNPs seen between two polymorph sequences, between A and B and between C and D. Nucleotides outlined in bold are those responsible for the amino acid substitution. Fhep numbers seen to the left of the nucleotide sequences correspond to individual qlk numbers used to distinguish between ESTs in the *F. hepatica* EST database. The three chosen qlk numbers for each sequence presented here are from the top three hits identified when locally BLASTing the *F. hepatica* EST database yet are representative of all sequences matching each peptide amino acid sequence.

The MSMS tryptic fragments strategy was designed to delineate the Cat L protease superfamily but also identified two non-synonymous single-nucleotide polymorphisms (nsSNPs) that ultimately gave rise to single amino acid polymorphisms (SAAPs) (Figure 4). Firstly, the peptide sequence characteristic of CL1 Cat L proteases, VTGYYTVHSGSE**V**ELK (Figure 4 sequence A), was observed in spot 18A, identified as CL1B (CAC12806), as well as the alternate sequence VTGYYTVHSGSE**A**ELK (Figure 4 sequence B) within the same spot (replicated in spot 29, see Table S3). This nsSNP shows a nucleotide switch from a thymine to a cytosine, creating a conservative amino acid switch, from a valine residue to an alanine. Sequence A was identified on eleven occasions (including twice in spot 19 and three times in spot 11) where as sequence B was only located in three spots. Analysis of ESTs within our in house translated database of *F. hepatica* ESTs (available at ftp://ftp.sanger.ac.uk/pub/pathogens/Fasciola/) revealed a total of 125 matching sequences equivalent to sequence A. In contrast, for sequence B, only eight matching EST sequences were identified. As a result, sequence B has an estimated minor allele frequency of 6.4%, based on the entries currently within the EST database.

A second potential nsSNP was identified showing an amino acid switch from a polar threonine residue to a basically charged arginine residue creating a tryptic cleavage site (Figures 3B and 4). Underlying this amino acid switch was a nucleotide substitution from a cytosine to a guanine. Analysing the in house database, a total of 118 ESTs were found to match QFGLETESSYPY**T**AVEGQCR (Figure 4 sequence C) and 30 ESTs that match to the sequence QFGLETESSYPY**R** (Figure 4 sequence D). Therefore, this amino acid change, producing sequence D, has an estimated minor allele frequency of 25.4%. Both sequences, C and D, were found in spots 13, 17 and 20. Interestingly, wherever the novel sequence VTGYYTLHSGNEAGLK was located, the peptide QFGLETESSYPYR resulting from a nsSNP creating the arginine residue, was always present. However, the reverse was not seen.

### EST Cathepsin L Protease

**Figure 5:**
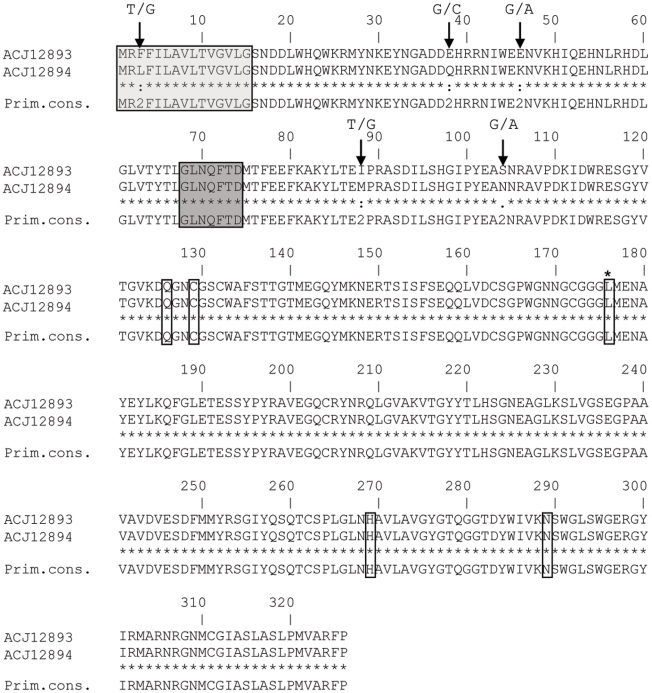
Novel CL1D protease sequences. Boxed and shaded in light grey are the predicted signal peptides using SignalP 3.0. Boxed and shaded in dark grey is the conserved GXNXFXD motif for autoactivation. Arrowed are the five amino acid substitutions varying between both contigs with the associated nucleotide substitutions above each. Individual boxed amino acids correspond to the active site residues with the exception of one, labelled with a *. This corresponds to the leucine at position 69 (papain numbering) dictating substrate specificity [61], [62]. The dashed line indicates the start of the N-terminus of the mature enzyme. A primary consensus sequence (Prim.cons.) is also included. Alignment was performed using ClustalW [63].

Two, full length, Cat L protease contigs were constructed from the matching ESTs to further characterise the novel peptides identified during MSMS analysis (Figure 5). This gave rise to two sequences 99.0% similar in nucleotide sequence (see Figure S2) and 98.5% similar in amino acid sequence. This corresponded to 10 nucleotide changes with only 5 of these resulting in amino acid substitutions. One of these substitutions occurred within the signal peptide but was still confirmed as a predicted signal peptide. The other four changes found, occurred within the pro-peptide segment, with the highly conserved auto-activation motif GXNXFXD unaltered. Both sequences, when compared at the amino acid level, showed 78.2% sequence identity (87% comparing nucleotides) to the previously described secreted cathepsin L2 (AAC47721). However, minor variations were observed when compared to secreted cathepsin L1 (AAB41670, clade CL1A), showing 92.9% (EU835857, 95% using nucleotides) and 94.5% (EU835858, 96% using nucleotides) sequence similarity.

The new sequences share 100% identity with CL1B members from analysis of the S2 subsite residues determined by Turk *et al.* [37], namely amino acid residues 67, 68, 133, 157, 158, 160 and 205 (Papain numbering). However, there is variation in 3 of the 5 mutation hotspots defined by Irving *et al.* [23] of which the majority are in hotspots I and II. Residues 156^(263)^, 158^(265)^ and 159^(266)^ in hotspot I, residues 66^(173)^, 79^(186)^ and 91^(198)^ in hotspot II and residue 173^(280)^ in hotspot III (*Fasciola* numbering [Numbers in superscript correspond to their position in Figure 5]) share no homology with CL1B members. However, many show homology to CL1A members including all three residues from mutation hotspot II and the solitary residue in hotspot III.

The novel sequences were passed through a Kolaskar and Tongaonkar Antigenicity prediction method [34] to identify potential antibody epitopes. Interestingly, an epitope identified in CL1A and CL1B members (Figure 5: residues 199 to 236) was now split into two smaller epitopes spanning residues 199–222 and 229–236 as a result of a maximum of 3 sequence variations (Figure 5: residues 225, 227 and 228).

### Phylogenetic Analysis

**Figure 6:**
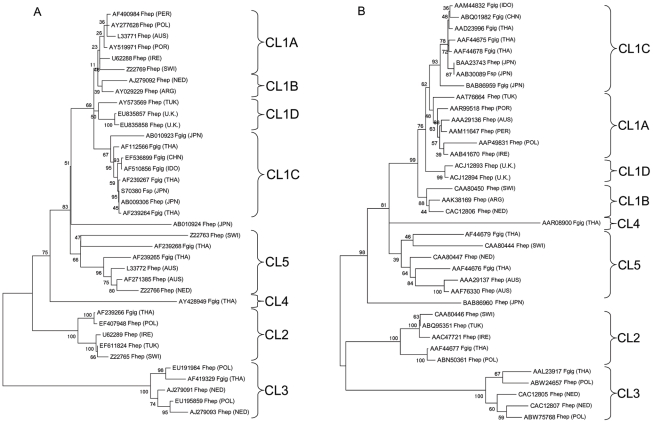
Phylogenetic analysis of the *Fasciola* Cat L family. Phylogenetic trees constructed using *F. hepatica* and *F. gigantica* Cat L protease nucleotide and amino acid sequences. All reported accession numbers are from Genbank with the suffix Fhep for *F. hepatica* and Fgig for *F. gigantica*. The origin of each Cat L sequence are reported in parentheses (ARG – Argentina, AUS – Australia, CHN – China, IDO – Indonesia, IRE – Ireland, JPN – Japan, NED – The Netherlands, PER – Peru, POL – Poland, POR – Portugal, SWI – Switzerland, THA – Thailand, TUK – Turkey and U.K. – United Kingdom). A) A neighbour-joining tree using nucleotide data constructed through MEGA v 4.0 with 1000 trial bootstrapped support using a Nei-Gojobori/Jukes-Cantor calculation. B) Neighbour-joining phylogenetic tree constructed using amino acid sequences through MEGA v 4.0 with 1000 bootstrapped support and a Poisson correction.

The objective of a phylogenetic analysis was to identify the origins of our newly identified Cat L protease sequences and to assess the overall clade structure of the *Fasciola* Cat L protease sub-family. Phylogenetic trees were constructed using nucleotide and amino acid data separately in order to delineate the phylogenetic relationship of the *Fasciola* Cat L proteases. This strategy produced trees of high similarity (Figures 6A, S3 and S4) providing high levels of confidence when assessing the overall *Fasciola* Cat L protease relationship.

As with previous studies our Trees divided the Cat L proteases into 5 distinct clades [23], [38]. The NEJ specific CL3 proteases and the adult CL2 and CL5 proteases formed three of these five clades with strong bootstrap support. The public entry AY428949 from *F. gigantica* juveniles, did not cluster with any other Cat L protease in our analyses and forms a second juvenile clade termed CL4 [24]. The fifth clade encompasses the CL1 proteases where extensive sub-division has previously been identified; in our study, CL1 being split into 4 sub-clades. The *F. hepatica* Cat L1 proteases have undergone much expansion, as would also be expected in *F. gigantica*, and as a result of the greater number of deposited cDNAs in the public domain three of the 4 sub-clades consist entirely of *F. hepatica* entries; namely CL1A, CL1B and a new sub-clade CL1D. The novel polymorphs identified in the present study clustered together along with AY573569, previously classified as CL1A, forming the new CL1D clade. The remaining CL1 clade, CL1C, contains *F. gigantica* entries only, although one Japanese entry, classified as *F. hepatica*, is also included; however this is most likely to be a hybrid species [38]. There was much variation with the positioning of AB010924, which may indicate a possible sixth clade. However, further entries may be required or genomic sequencing to confirm this finding.

Analysis of the Cat L protease amino acid sequences produced trees that were closely similar to one another and to those produced using nucleotides, providing a further level of confidence in the trees produced (Figures 6B, S5 and S6). As with nucleotide data, the Cat L proteases could be divided into 5 distinct clades. The NEJ CL3 and CL4 clades and adult CL2 and CL5 clades were resolved as previous, providing a high degree of certainty. The CL1 clades clustered together, as prior analyses, yielding CL1A, CL1B, CL1C and the novel CL1D. CL1D contains the new isoforms outlined in the present study but without the CL1 protease ATT76664 (Nucleotide AY573569) supported with strong bootstrap support (77% Minimum Evolution, 76% Neighbour Joining and 78% Maximum Parsimony).

Both our phylogenetic and proteomic studies supported one another regarding the clade structure of the Cat L protease family in *Fasciola*. As with Robinson *et al.* [38] only members of adult *F. hepatica* Cat L protease clades could be identified during 2-DE analysis, namely CL1A, CL1B, CL2, CL5 and the new CL1D. Supporting the *in vitro* study of Robinson *et al.* [38], the ES Cat L proteases were predominantly made up of CL1 proteases (71.68% *in vitro* and 72.78% *in vivo* from this clade). ES Cat L proteases from the novel CL1D sub-clade appeared to be expressed at levels similar to the CL1B sub-clade constituting 10.34% and 9.11% of the overall protease content *in vitro* and *in vivo* respectively (Table S1). Previously, the CL1B clade constituted 32.09% *in vitro* [24], which approximates to the combined CL1B and CL1D clades along with those NFD (29.47% *in vitro* and 36.80% *in vivo*). The remaining Cat L proteases originate from the CL2 clade (21.51% *in vitro* and 15.07% *in vivo*) and the CL5 clade (5.52% *in vitro* and 5.61% *in vivo*).

## Discussion

The evolutionary success of *Fasciola hepatica* is, in part, due to its adaptability to successfully invade and establish in different mammalian hosts [23]. The invasion of multiple host species is supported by the secretion of the multifunctional and multi-family member Cat L proteases within the host environment. Cat L proteases, stored as inactive zymogens [39], are released in relatively large quantities [0.5–1 µg/adult/hour: 40] in order to facilitate obligate blood feeding [41]; often degrading 1.5×10^8^ red blood cells h^−1^ worm^−1^ [41].

The present study demonstrates that comprehensive high resolution 2-DE mapping of these ES Cat L proteases using narrow and micro range IPG strips and large format SDS-PAGE resolves many issues derived from reductionist based experiments. In support of previous studies [24] we have 1) identified 3 of the 5 Cat L clades (CL1, CL2 and CL5) in *Fasciola* species from adult liver fluke, of which the CL1 and CL2 clades are the major constituents 2) failed to identify *F. _igantic* CL1 representatives and 3) failed to identify CL3 and CL4 representatives, juvenile specific and enhancing the belief that they are important in gut invasion [42]. No other proteases were identified during this study highlighting the sole reliance of *F. Hepatica* on these proteases to provide nutrition.

In addition, the current study has also revealed differences in the Cat L protease complement from the artificial *in vitro* biology platform of liver fluke ex-host, and liver fluke within the natural host, *in vivo*. A novel Cat L CL1 clade isoform has also been identified along with the first report of single amino acid polymorphism (SAAP) identified via experimental non-model organism proteomic investigations.

Previous proteomic studies have encountered difficulties in Cat L protease identification in the non-genome sequenced *F. Hepatica* using PMF [14], [15]. MSMS peptide sequencing allowed for a more robust analysis of the Cat L proteases [the present study and 38]. However, although all sequences were confirmed as Cat L proteases and assigned to the appropriate clade, not all sequences could be firmly linked to a specific sub-clade or database entry. This is directly related to the large degree of allelic diversity observed between Cat L proteases produced by *F. Hepatica* [43], especially with the onset of triploidy in Fasciolids [44]. The full extent of Cat L protease diversity will require a significant high-throughput sequencing effort of natural populations of liver fluke.

Thus, with new confidence in assigning Cat L proteases to a specific clade (CL1, CL2 or CL5) the Cat L protease expression profiles from both *in vitro* and *in vivo* preparations could be robustly assessed. The assays revealed there were differences between the two preparations with respect to the presence or absence of protein spots and the regulation of protein spots. Key changes between *in vitro* and *in vivo* Cat L proteases included the addition of four abundant protein spots (Spots 5 and 7 – CL2, Spots 19 and 20 – CL1A or B) within the *in vitro* profile (and a further 7 CL1 lower abundant proteins). All four of these proteins were identified in other locations within the *in vitro* ES proteome profile, which suggests they are post translationally modified (PTM).

The most likely PTM seen on Cat L proteases will be related to mannose 6-phosphate phosphorylation, signalling transport to the lysosomes. The residues important for lysosomal targeting, via phosphorylation, in human Cat L proteases are partially conserved in *Fasciola* sp. [45] suggesting PTM of *Fasciola* Cat L proteases via phosphorylation is a likely candidate. As these parasites are cultured ex-host, post translational phosphorylation may indicate an increase in Cat L protease secretion in response to a poor nutritional environment or may represent modification related to the chemical environment. While the overall biochemical activity of the cat L proteases secreted *in vitro* and *in vivo* may be similar with model and calculated natural substrates (see Table S1), the potential finding of additional PTM in the ex-host preparations warrants further investigation to reveal their influence on both masking enzymatic activity and future Cat L/protein interactions. However, PTM identification might be a reflection of plasticity of the host-parasite in changing host environments.

A significant increase in the abundance of Cat L protease from the CL1 clade was observed in preparations derived from *in vivo* treatments (Protein Spot 12). All the remaining changes in expression between *in vitro* and *in vivo* samples, although not confirmed significant (further sampling required), were also identified as CL1 members. Within the host, *F. Hepatica* is involved in interactions between both host and parasite which are clearly absent ex-host *in vitro* culture. Therefore, it is possible that the variability in expression of these CL1 clade Cat L proteases may represent a specific response of the parasite to the host environment, such as immune evasion and nutrient acquisition [41], [46]. The selection pressure exerted by the host on the CL1 Cat L proteases and the plasticity of CL1 expression in *F. Hepatica* has most likely led to the divergence seen in the CL1 clade producing the repertoire of sub-clades seen in the present study. The main challenge faced by *F. Hepatica in vitro* culture and within the host bile ducts is nutrient acquisition. As the CL1 clade is most likely responsible for the degradation of host haemoglobin for nutritional requirements [47], the variation in CL1 expression seen in the present study will be in response to nutritional acquisition. With lower risks of host immune attack in bile [48], [49] and the reduced need to migrate through the interstitial matrices the requirement to vary the regulation of CL2 and CL5 clade Cat L proteases in these 2 systems (*in vitro* and *in vivo*) may have become less important.

The selection pressure exerted on the CL1 clade by the host is highlighted by two further aspects of this study, namely the discovery of a new sub-clade CL1D and the discovery of SAAPs in the CL1 clade. Using contig sequences derived from the novel Cat L EST sequences, a 92.9–94.5% amino acid sequence identity to CL1A was shown (78.2% to CL2 84–85% to CL5), suggesting these novel Cat L sequences are CL1 members. In addition, they appear not to be the potentially novel Cat L protease sequences described by Robinson *et al.* [42]. Following phylogenetics it appears the new Cat L protease sequences constitute a novel CL1 sub-clade, CL1D. This clade appears to have initially diverged with the rest of the CL1 members following the division of the CL5 clade. The separation of this clade also seems to be an early divergence post-division of *F. Hepatica* and *F. Gigantic* CL1 members. Additionally, it appears this clade has branched from the CL1B clade, highlighted by the clustering of a previously classified CL1B (AY573569) with the two CL1D sequences at the nucleotide level but not at the protein level, suggesting they are now functionally different.

A comparative analysis of the S2 active site residues in the substrate binding region [50] predicts that the new CL1D would be biochemically identical to CL1B, and thus not support the phylogenetic analysis of a new sub-clade. However, due to significant variations in 2 of the 5 mutation hotspots identified by Irving *et al.* [23] (mutation hotspots I and II, one on either side of the active site cleft) relative biochemical activity would need to be confirmed. Three amino acids under positive selection pressure in each of the two mutation hotspots vary between CL1B members and CL1D members. Both hotspots are suggested to be involved in interactions with substrates or other proteins outside of the normal binding regions [23]. In addition, amino acids 156 and 159 (*Fasciola* numbering) in hotspot I are suggested to be involved in S2 sub-site interactions [37] and may well influence biochemical activity.

An additional variation between CL1B and CL1D members can be found in mutation hotspot III, the hotspot found on the edge of the R-domain of the cat L proteases [23]. The exact function of hotspot III, and IV and V, are unclear but suggested to be involved in proteolytic interactions with globular proteins [23]. It appears that CL1D members have an intermediary biochemical activity between CL1A and CL1B members as they share similarities with both clades. Therefore, until confirmation of the biochemical activity of CL1D members, and additionally CL1B members, has been confirmed in relation to CL1A members, it seems warranted to keep the CL1D members in a separate sub-clade of the CL1 proteases.

The second aspect of variation seen in the CL1 clade relates to the discovery of two nsSNPs, including one variant in the newly identified CL1D sub-clade. This is the first report of single amino acid polymorphism (SAAP) identified via experimental non-model organism proteomic investigation as population genetics and genomic discovery approaches are the usual methods for SAAP identification [51]. This finding highlights the power of gel based proteomics to reveal differences at the amino acid substitution level.

The first SAAP, position 120 (*Fasciola* numbering), involved a conservative amino acid switch, from a valine residue to a alanine residue, both non-polar. This SAAP had a low estimated minor allele frequency of 6.4% and therefore, was only located in three Cat L proteases spots (18A, 28 and 29, all CL1 members). However, the second SAAP was a switch from a small, polar threonine residue to a large polar, positively charged, arginine residue. This second switch was shown by Irving *et al.* [23] to be an amino acid residue (position 91 *Fasciola* numbering) under positive selection pressure increasing the frequency of this substitution and, accordingly, had an approximate minor allele frequency of 25%. This particular SAAP was located in eight protein spots, all CL1 members, and demonstrates the increased frequency when compared to SAAPs not subjected to selection pressures. Only one variant of this T91R SAAP was located to the novel cat L1D protease identified in this study, further suggesting divergence from the CL1B clade.

Although outside of the extended Cat L protease active site the T91R substitution could have potential effects on the function of these enzymes. This particular SAAP site (site 91 *Fasciola* numbering) is located in a mutation hot spot (II) and forms one side of the active site cleft. As mentioned, this region may be involved in interactions with substrates outside of the normal binding regions [23] and may therefore have implications in the specificity of higher order interactions in the enzymes that carry this SAAP. However, this will need to be confirmed.

Both SAAPs observed in expressed Cat L proteases in the present study may confer structural alterations that could affect recognisable epitopes and, if used as a chemotherapeutic target, drug binding sites [52]. Therefore, these SAAPs provide an opportunity to study antigenic variants which may be useful for future development of control measures. Additionally, they lend themselves to modelling based studies to ascertain any conformational alterations such as solvent exposure and interactions.

The plasticity revealed in the CL1 clade of Cat L proteases may impact on the future development of vaccines based on this target. Will a vaccine targeted towards a CL1 member effectively overcome the antigenic diversity seen in this clade? It has been postulated that significant economic benefit would arise from vaccination resulting in a reduction of worm burden of >50% [53], [54]. Other vaccine trials would favour formulating vaccines based on CL2, CL3 or CL5 members. Although direct comparisons between vaccine trials are difficult to perform there appears strong evidence that trials with Cat L protease from CL2, CL3 and CL5 clades, individually and in combination with other antigens, are more promising in relation to worm burdens than CL1 trials. Recent trials with the NEJ clade CL3 have produced early success reaching reductions of 52% after vaccination with only this Cat L protease [55]. Successful early trials with the CL5 clade have also been performed providing a 51.4% reduction using the Cat L protease alone and a substantial 83% reduction when used in combination with Cat B [56]. CL2 trials have been more extensive than with CL3, CL4 or CL5 members and reported protection ranges from 33–60% using CL2 alone [57] but in conjunction with fluke haemoglobin have reached 72.4% [58]. Trials involving CL1 have been by far the most studied with protection beginning at 0% and reaching a maximum of 69.5% [58], with the majority consistently below the recommended 50% reduction.

The plasticity within the CL1 clade may be underpinning the observed variability in previous vaccine trials using CL1 proteases. Others have shown that SAAPs can have a profound effect on the antigenicity of pathogenic organisms [59]. Patterns of excessive polymorphisms in parasitic antigens are consistent with high selection pressure and are suggested to function in immune evasion [28]. Furthermore, Irving *et al.* [23] identified excessive polymorphism in 5 mutation hotspots (previously discussed) which may be affecting interactions with immune effector molecules [23], [59]. The expansion of the *F. Hepatica* CL1 clade into 3 sub-clades (1A, 1B and 1D) could be a direct effect of immune selection. Indeed, the novel CL1D isoforms identified in this study show altered predicted epitopes from CL1A and CL1B members as a result of three SAAPs splitting a large epitope into 2 of smaller size. However, it has been shown that a single SAAP can be responsible for altering the immune recognition of parasitic antigens [28]. The evidence presented in the current study, showing further expansion of the CL1 clade, in addition to SAAPs only identified in CL1 members enhances the possibility that this plasticity underpins the variability of protection seen in vaccine trials. CL1 trials have already shown potent anti-embryonation effects and significant reductions in fecundity [58]. This raises the possibility of formulating improved combinations of Cat L proteases to significantly reduce worm burden and fecundity/embryonation in tandem by robust population proteomics assays [60].

This study has also raised the possibility of a sixth Cat L protease clade. The Cat L protease (Accession number AB010924) was always placed singularly and did not cluster with any other members. In prior analyses this entry has been classified as a CL2 member. In the present study, AB010924 clustered in varying positions between CL5 and CL1, off the CL1 clade or near CL2 and CL5. Further investigation or enhanced genomic information will be needed to confirm this finding.

To summarise, comparison of the ES product sub-proteomes has highlighted variations in the Cat L protease profile between ex-host artificial platforms and direct *in vivo* assays most likely related to PTM. Therefore, *in vitro* studies on the Cat L proteases from Fasciolids may increase the understanding of host-parasite relationships by revealing potential plasticity of an important vaccine target superfamily. In this case study, the plasticity of Cat L protease expression has been shown to be limited to the CL1 clade, leading to the discovery of a new CL1 sub-clade revealed through proteomic-EST sequencing-phylogenetic studies. For the first time, this study has identified experimentally single amino acid polymorphisms (SAAP) in a key immunotherapeutic parasite target. Gel based proteomics of pooled samples from populations should be considered for SAAP based biomarker discovery. To effectively formulate a vaccine based on the Cat L proteases we suggest that discovery programmes focus on an alternate Cat L protease clade, such as CL5 where promising early results have been shown [56].

## Supporting Information

Figure S1 Sequence alignment of six *F. hepatica* cathepsin L1 protease sequences from the CL1A and CL1B sub-clades commonly hit during MSMS analysis and designated as NFD. Boxed sequence indicate amino acid variation between these six sequences in the mature enzyme only. Boxed shaded regions indicate peptides commonly sequenced via MSMS including the N-terminal peptide AVPDKIDWR. The dotted boxed region indicates a peptide sequenced only on two occasions during MSMS. The amino acids labelled with a * locate the site of a SAAP identified using MSMS and a translated EST database.
(2.24 MB TIF)

Figure S2 Nucleotide contigs of two clones of a novel cathepsin L protease from the CL1D sub-clade identified in the present study. Variations between the two contigs are in bold red type.
(2.31 MB TIF)

Figure S3 A) Phylogenetic tree constructed using *F. hepatica* and *F. gigantica* cat L protease nucleotide sequences. A maximum parsimony tree using nucleotide data constructed through MEGA v 4.0 with 1000 trial bootstrapped support. All reported accession numbers are from Genbank B) Phylogenetic tree constructed using *F. hepatica* and *F. gigantica* cat L protease nucleotide sequences. A minimum evolution tree using nucleotide data constructed through MEGA v 4.0 with 1000 trial bootstrapped support using a Kimura 2-parameter model. All reported accession numbers are from Genbank.
(0.66 MB TIF)

Figure S4 A) Phylogenetic tree constructed using *F. hepatica* and *F. gigantica* cat L protease amino acid sequences. A maximum parsimony tree using amino acid data constructed through MEGA v 4.0 with 1000 trial bootstrapped support. All reported accession numbers are from Genbank B) Phylogenetic tree constructed using *F. hepatica* and *F. gigantica* cat L protease amino acid sequences. A minimum evolution tree using amino acid data constructed through MEGA v 4.0 with 1000 trial bootstrapped support using a Kimura 2-parameter model. All reported accession numbers are from Genbank.
(1.31 MB TIF)

Figure S5 A) Full size, un-cropped, representative 2-DE protein arrays of *in vitro* ES Cat L proteases as seen in the main manuscript. The dotted region indicates the area shown within the main manuscript. B) Full size, un-cropped, representative 2-DE protein arrays of *in vivo* ES Cat L proteases as seen in the main manuscript. Protein spots from around the 30 kDa marker were taken for MSMS analysis based on previous work and identifications within our laboratory (Morphew *et al.* 2007 MCP 6 963–972) indicating the only addition of *F. hepatica* protein to host bile was located in this region. The dotted region indicates the area shown within the main manuscript.
(1.78 MB TIF)

Figure S6 A) MSMS sequence analysis using peptide sequencer (MassLynx v. 5.0, Micromass, UK) from the fragmentation of a precursor ion m/z 1224.63 (2+) representative of cathepsin L clade 2. Interpretation of the y and b ion series provided the peptide sequence NQGQCGSCADAFSTTGAVEGQFR. B) MSMS sequence analysis using peptide sequencer (MassLynx v. 5.0, Micromass, UK) from the fragmentation of a precursor ion m/z 849.86 (2+) representative of cathepsin L clade 2. Interpretation of the y and b ion series provided the peptide sequence ASASFSEQQLVDCTR.
(0.81 MB TIF)

Figure S7 A) MSMS sequence analysis using peptide sequencer (MassLynx v. 5.0, Micromass, UK) from the fragmentation of a precursor ion m/z 991.42 (2+) representative of cathepsin L clade 5. Interpretation of the y and b ion series provided the peptide sequence DAPAFMASLASVPMVAQFP. B) MSMS sequence analysis using peptide sequencer (MassLynx v. 5.0, Micromass, UK) from the fragmentation of a precursor ion m/z 724.85 (2+) representative of cathepsin L clade 5. Interpretation of the y and b ion series provided the peptide sequence FGLETESSYPYR.
(0.77 MB TIF)

Figure S8 A) MSMS sequence analysis using peptide sequencer (MassLynx v. 5.0, Micromass, UK) from the fragmentation of a precursor ion m/z 590.28 (3+) representative of cathepsin L clade 1. Interpretation of the y and b ion series provided the peptide sequence VTGYYTVHSGSEVELK. B) MSMS sequence analysis using peptide sequencer (MassLynx v. 5.0, Micromass, UK) from the fragmentation of a precursor ion m/z 580.90 (3+) representative of cathepsin L clade 1. Interpretation of the y and b ion series provided the peptide sequence VTGYYTVHSGSE*A*ELK including a single amino acids polymorphism (italicised).
(0.91 MB TIF)

Figure S9 A) MSMS sequence analysis using peptide sequencer (MassLynx v. 5.0, Micromass, UK) from the fragmentation of a precursor ion m/z 596.27 (2+) representative of cathepsin L clade 1. Interpretation of the y and b ion series provided the peptide sequence NSWGLSWGER. B) MSMS sequence analysis using peptide sequencer (MassLynx v. 5.0, Micromass, UK) from the fragmentation of a precursor ion m/z 550.29 (2+) representative of cathepsin L clade 1. Interpretation of the y and b ion series provided the N-terminal peptide of the mature enzyme sequenced as AVPDKIDWR.
(0.66 MB TIF)

Figure S10 A) MSMS sequence analysis using peptide sequencer (MassLynx v. 5.0, Micromass, UK) from the fragmentation of a precursor ion m/z 1007.06 (3+) representative of cathepsin L clade 1B. Interpretation of the y and b ion series provided the peptide sequence GNCGSCWAFSTTGTMEGQYMKNEK. B) MSMS sequence analysis using peptide sequencer (MassLynx v. 5.0, Micromass, UK) from the fragmentation of a precursor ion m/z 774.68 (3+) providing the identification of spot 12. Interpretation of the y and b ion series provided the peptide sequence YPYTAVEGQCR.
(0.95 MB TIF)

Figure S11 MSMS sequence analysis using peptide sequencer (MassLynx v. 5.0, Micromass, UK) from the fragmentation of a precursor ion m/z 748.86 (2+) providing the identification of spot 32. Interpretation of the y and b ion series provided the peptide sequence SGIYQSQTCSPLR.
(0.43 MB TIF)

Table S1 (0.01 MB PDF)

Table S2 (0.05 MB PDF)

Table S3 (0.13 MB PDF)
