# Supplementary figures and images for: Towards Delineating Functions within the Fasciola Secreted Cathepsin L Protease Family by Integrating In Vivo Based Sub-Proteomics and Phylogenetics
Source: PLoS Negl Trop Dis. 2011 Jan 4;5(1):e937. doi: 10.1371/journal.pntd.0000937 (PMC3014944; doi:10.1371/journal.pntd.0000937)

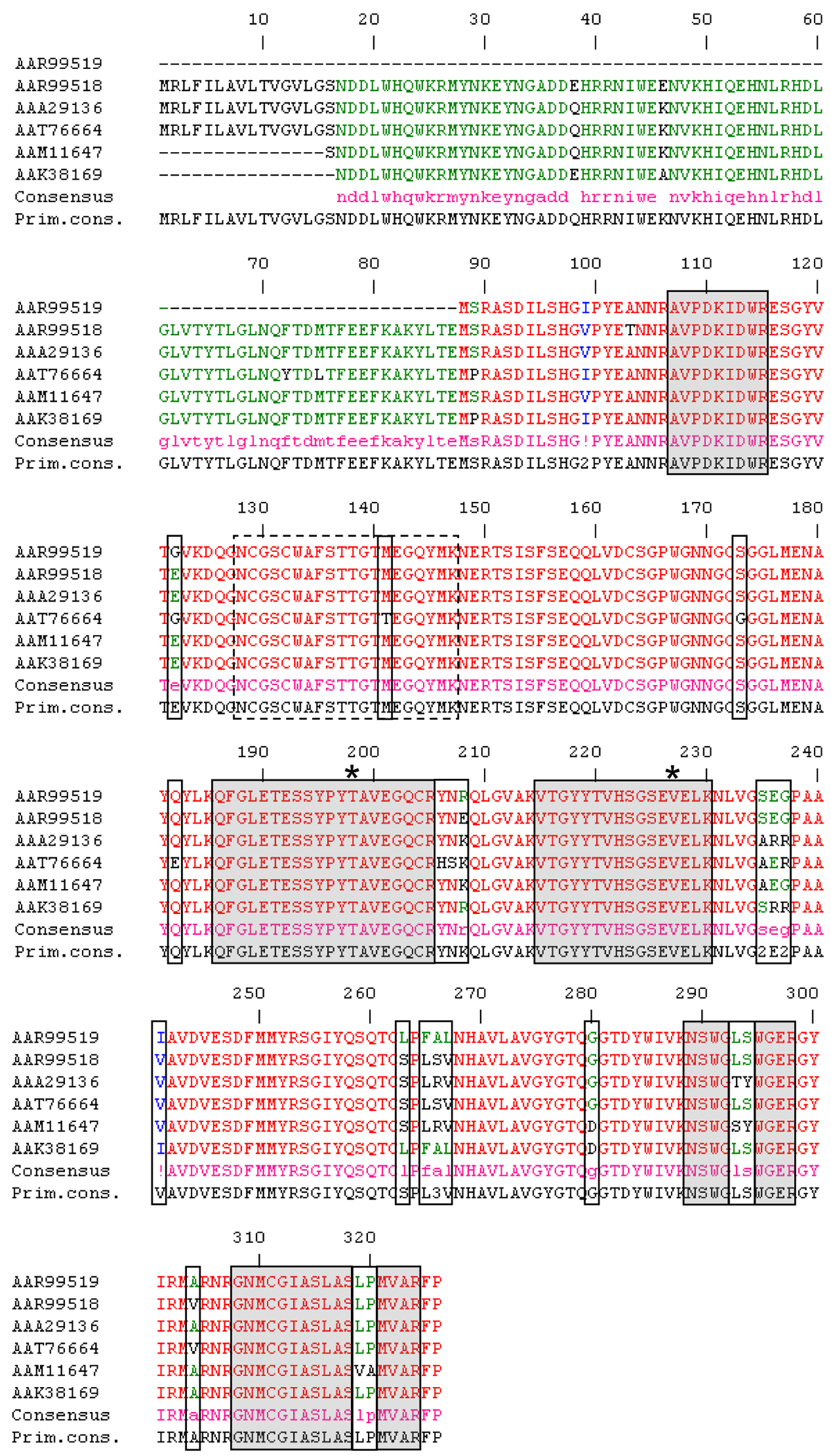

Supplement: Figure S1 — Sequence alignment of six F. hepatica cathepsin L1 protease sequences from the CL1A and CL1B sub-clades commonly hit during MSMS analysis and designated as NFD. Boxed sequence indicate amino acid variation between these six sequences in the mature enzyme only. Boxed shaded regions indicate peptides commonly sequenced via MSMS including the N-terminal peptide AVPDKIDWR. The dotted boxed region indicates a peptide sequenced only on two occasions during MSMS. The amino acids labelled with a * locate the site of a SAAP identified using MSMS and a translated EST database. (2.24 MB TIF) [file pntd.0000937.s001.tif]

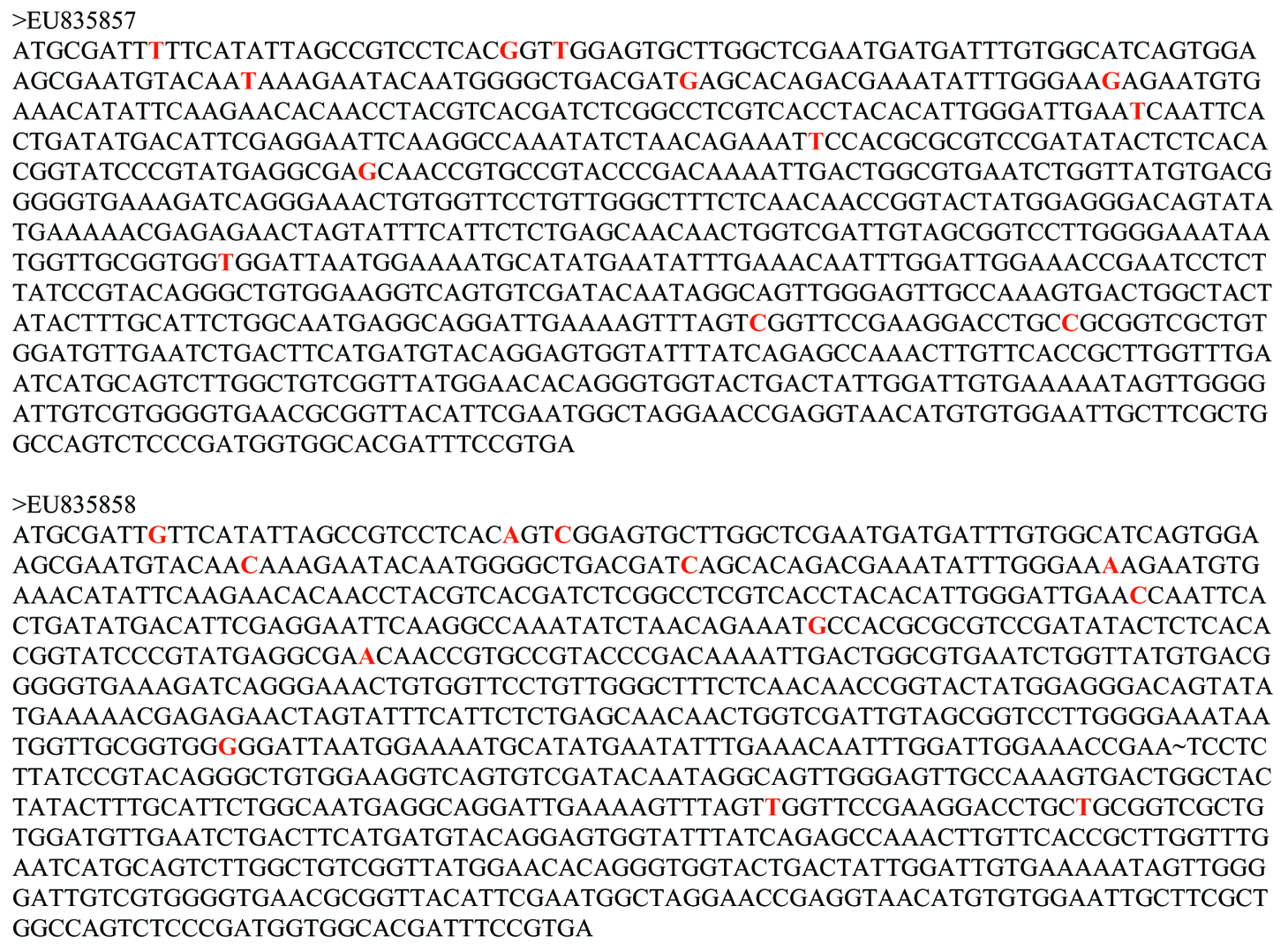

Supplement: Figure S2 — Nucleotide contigs of two clones of a novel cathepsin L protease from the CL1D sub-clade identified in the present study. Variations between the two contigs are in bold red type. (2.31 MB TIF) [file pntd.0000937.s002.tif]

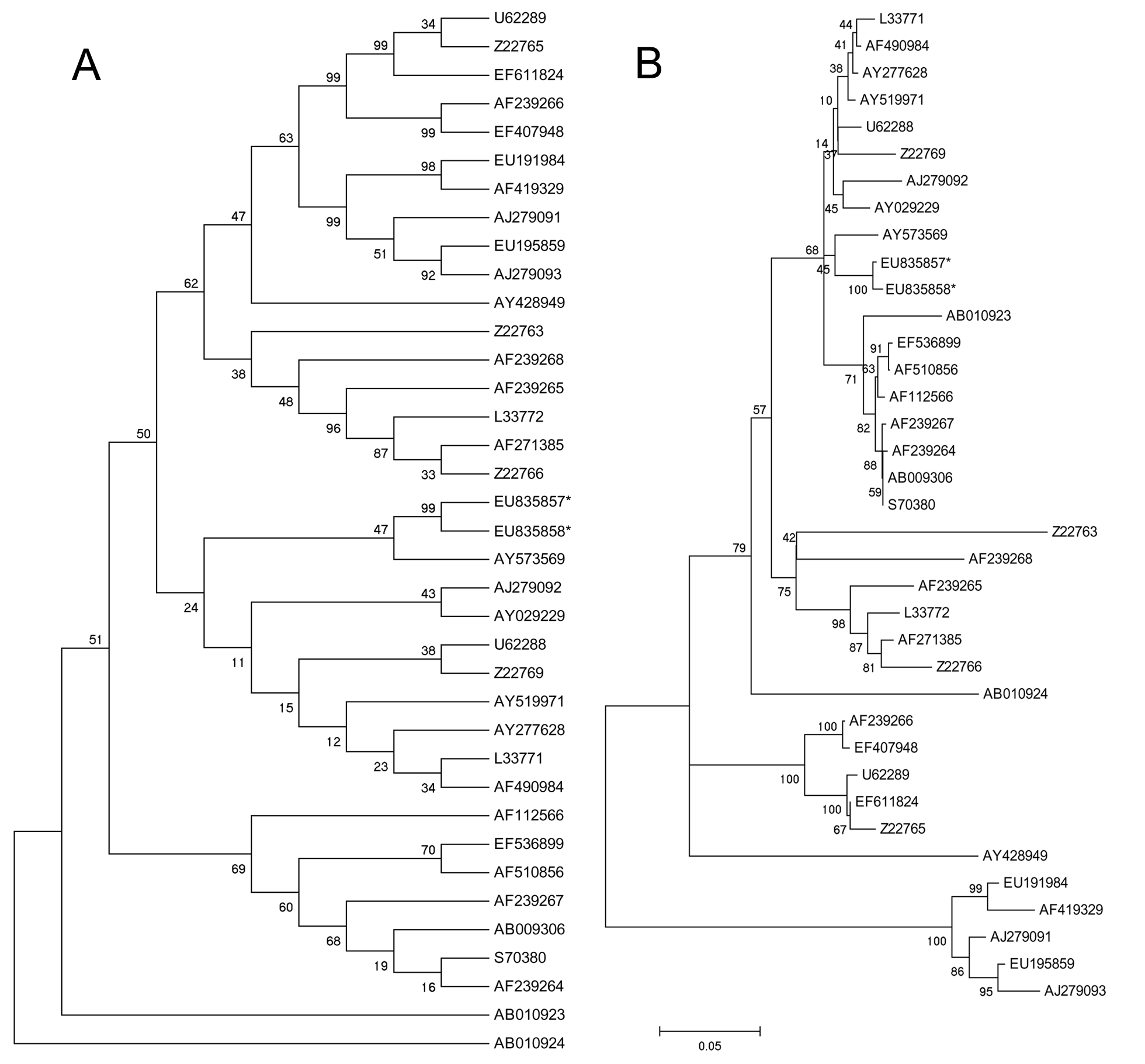

Supplement: Figure S3 — A) Phylogenetic tree constructed using F. hepatica and F. gigantica cat L protease nucleotide sequences. A maximum parsimony tree using nucleotide data constructed through MEGA v 4.0 with 1000 trial bootstrapped support. All reported accession numbers are from Genbank B) Phylogenetic tree constructed using F. hepatica and F. gigantica cat L protease nucleotide sequences. A minimum evolution tree using nucleotide data constructed through MEGA v 4.0 with 1000 trial bootstrapped support using a Kimura 2-parameter model. All reported accession numbers are from Genbank. (0.66 MB TIF) [file pntd.0000937.s003.tif]

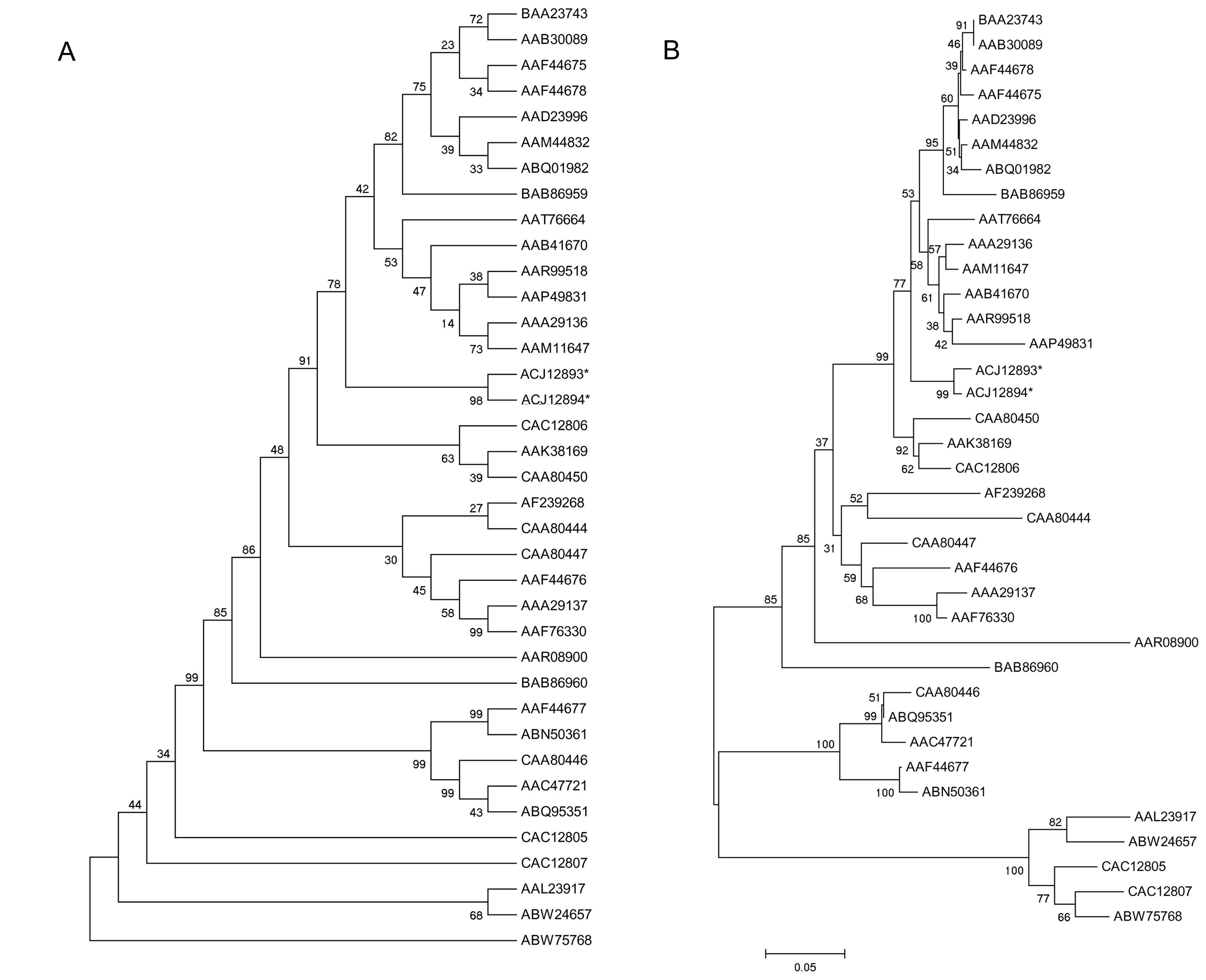

Supplement: Figure S4 — A) Phylogenetic tree constructed using F. hepatica and F. gigantica cat L protease amino acid sequences. A maximum parsimony tree using amino acid data constructed through MEGA v 4.0 with 1000 trial bootstrapped support. All reported accession numbers are from Genbank B) Phylogenetic tree constructed using F. hepatica and F. gigantica cat L protease amino acid sequences. A minimum evolution tree using amino acid data constructed through MEGA v 4.0 with 1000 trial bootstrapped support using a Kimura 2-parameter model. All reported accession numbers are from Genbank. (1.31 MB TIF) [file pntd.0000937.s004.tif]

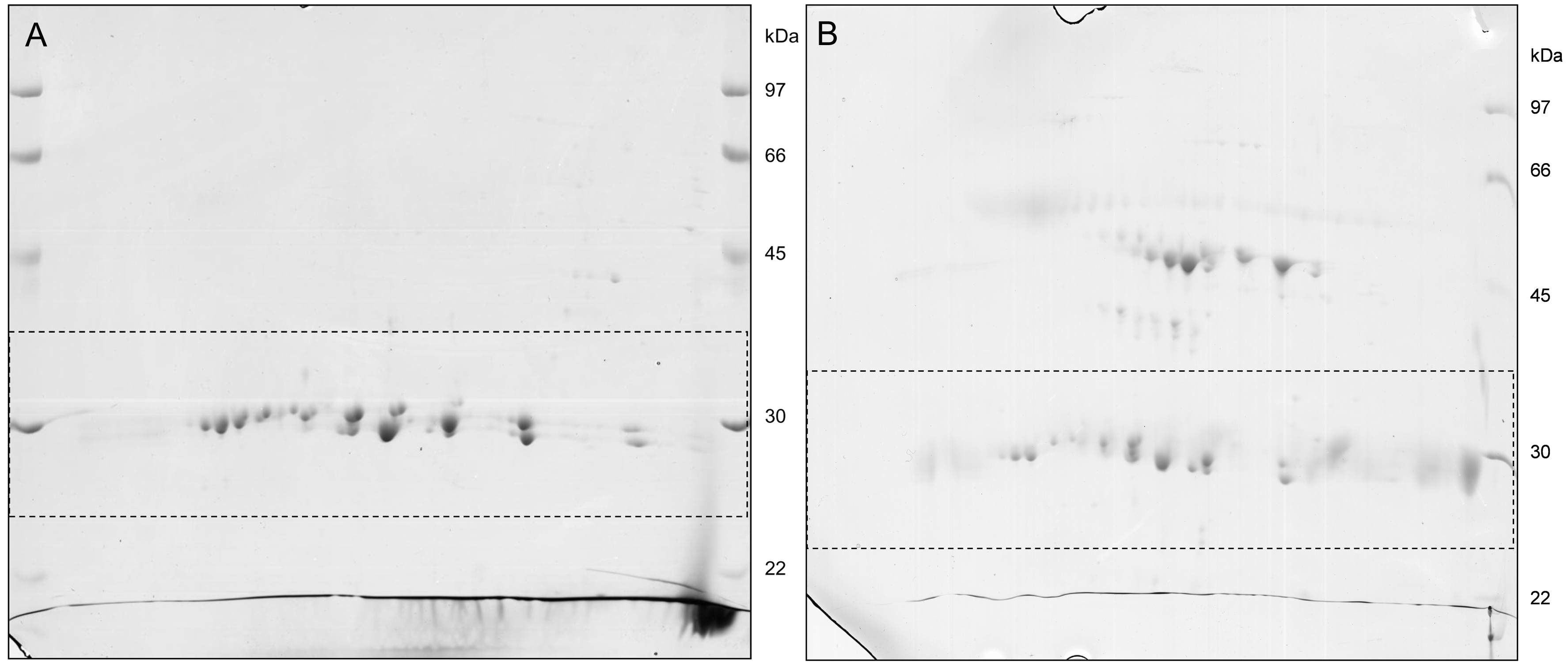

Supplement: Figure S5 — A) Full size, un-cropped, representative 2-DE protein arrays of in vitro ES Cat L proteases as seen in the main manuscript. The dotted region indicates the area shown within the main manuscript. B) Full size, un-cropped, representative 2-DE protein arrays of in vivo ES Cat L proteases as seen in the main manuscript. Protein spots from around the 30 kDa marker were taken for MSMS analysis based on previous work and identifications within our laboratory (Morphew et al. 2007 MCP 6 963–972) indicating the only addition of F. hepatica protein to host bile was located in this region. The dotted region indicates the area shown within the main manuscript. (1.78 MB TIF) [file pntd.0000937.s005.tif]

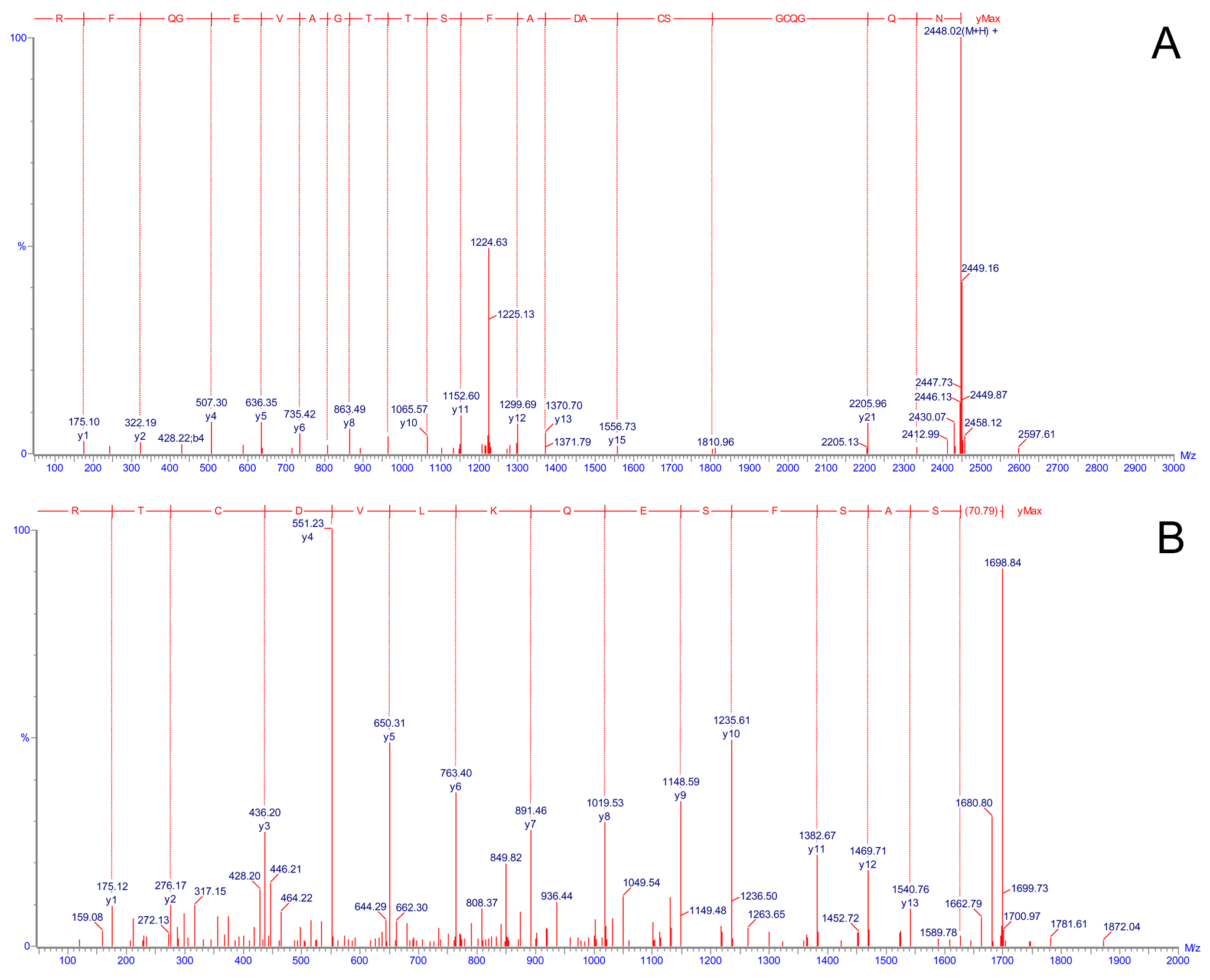

Supplement: Figure S6 — A) MSMS sequence analysis using peptide sequencer (MassLynx v. 5.0, Micromass, UK) from the fragmentation of a precursor ion m/z 1224.63 (2+) representative of cathepsin L clade 2. Interpretation of the y and b ion series provided the peptide sequence NQGQCGSCADAFSTTGAVEGQFR. B) MSMS sequence analysis using peptide sequencer (MassLynx v. 5.0, Micromass, UK) from the fragmentation of a precursor ion m/z 849.86 (2+) representative of cathepsin L clade 2. Interpretation of the y and b ion series provided the peptide sequence ASASFSEQQLVDCTR. (0.81 MB TIF) [file pntd.0000937.s006.tif]

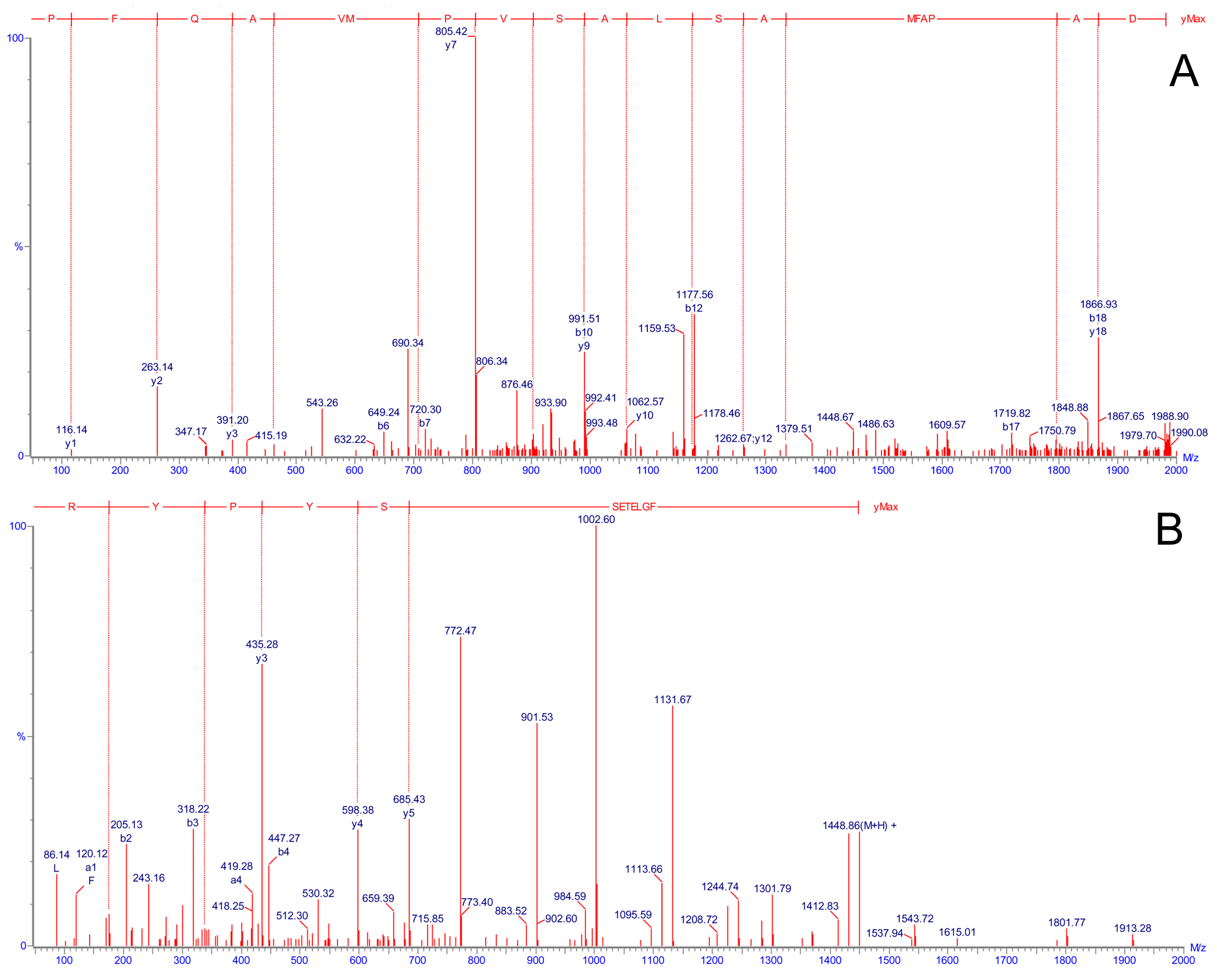

Supplement: Figure S7 — A) MSMS sequence analysis using peptide sequencer (MassLynx v. 5.0, Micromass, UK) from the fragmentation of a precursor ion m/z 991.42 (2+) representative of cathepsin L clade 5. Interpretation of the y and b ion series provided the peptide sequence DAPAFMASLASVPMVAQFP. B) MSMS sequence analysis using peptide sequencer (MassLynx v. 5.0, Micromass, UK) from the fragmentation of a precursor ion m/z 724.85 (2+) representative of cathepsin L clade 5. Interpretation of the y and b ion series provided the peptide sequence FGLETESSYPYR. (0.77 MB TIF) [file pntd.0000937.s007.tif]

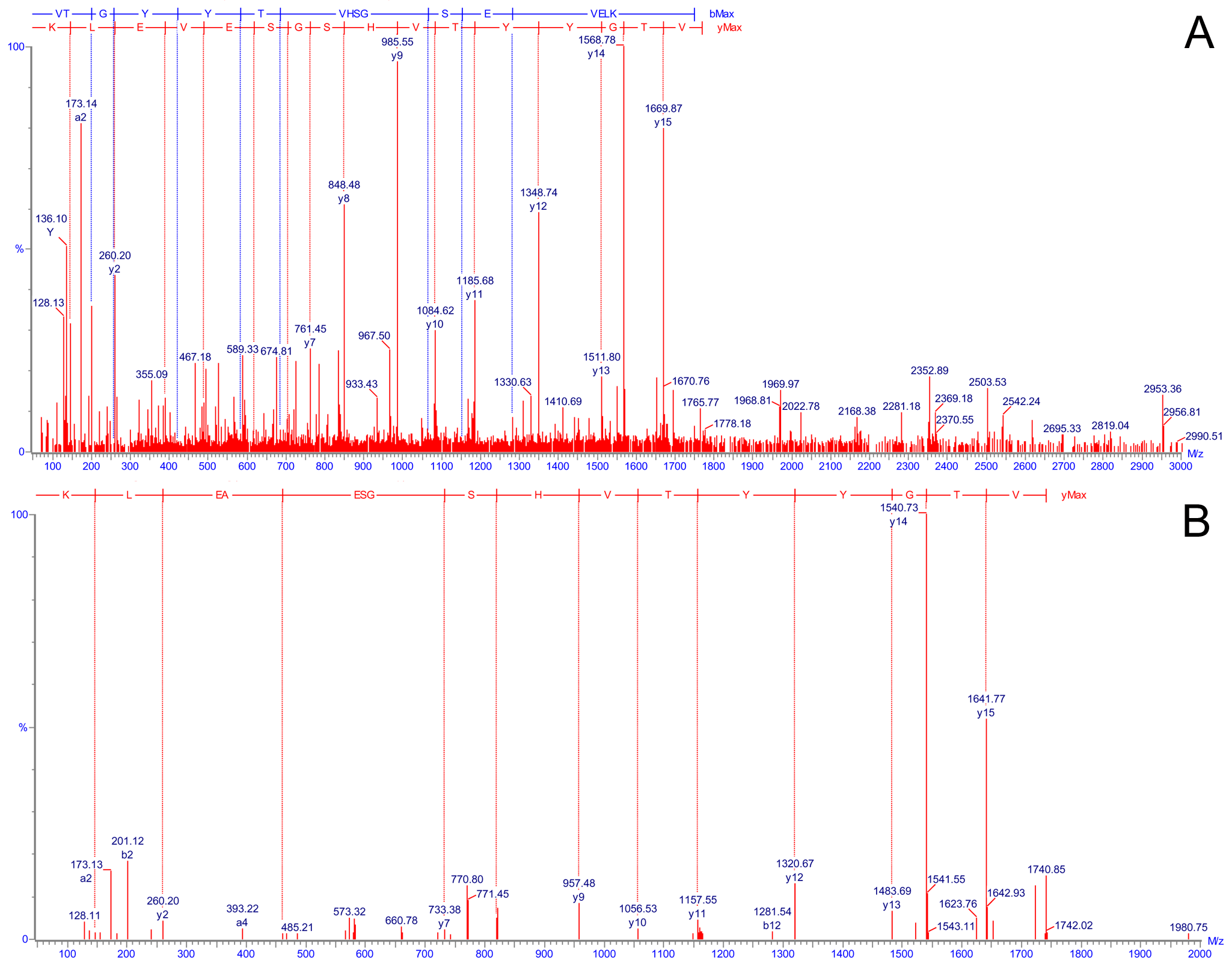

Supplement: Figure S8 — A) MSMS sequence analysis using peptide sequencer (MassLynx v. 5.0, Micromass, UK) from the fragmentation of a precursor ion m/z 590.28 (3+) representative of cathepsin L clade 1. Interpretation of the y and b ion series provided the peptide sequence VTGYYTVHSGSEVELK. B) MSMS sequence analysis using peptide sequencer (MassLynx v. 5.0, Micromass, UK) from the fragmentation of a precursor ion m/z 580.90 (3+) representative of cathepsin L clade 1. Interpretation of the y and b ion series provided the peptide sequence VTGYYTVHSGSEAELK including a single amino acids polymorphism (italicised). (0.91 MB TIF) [file pntd.0000937.s008.tif]

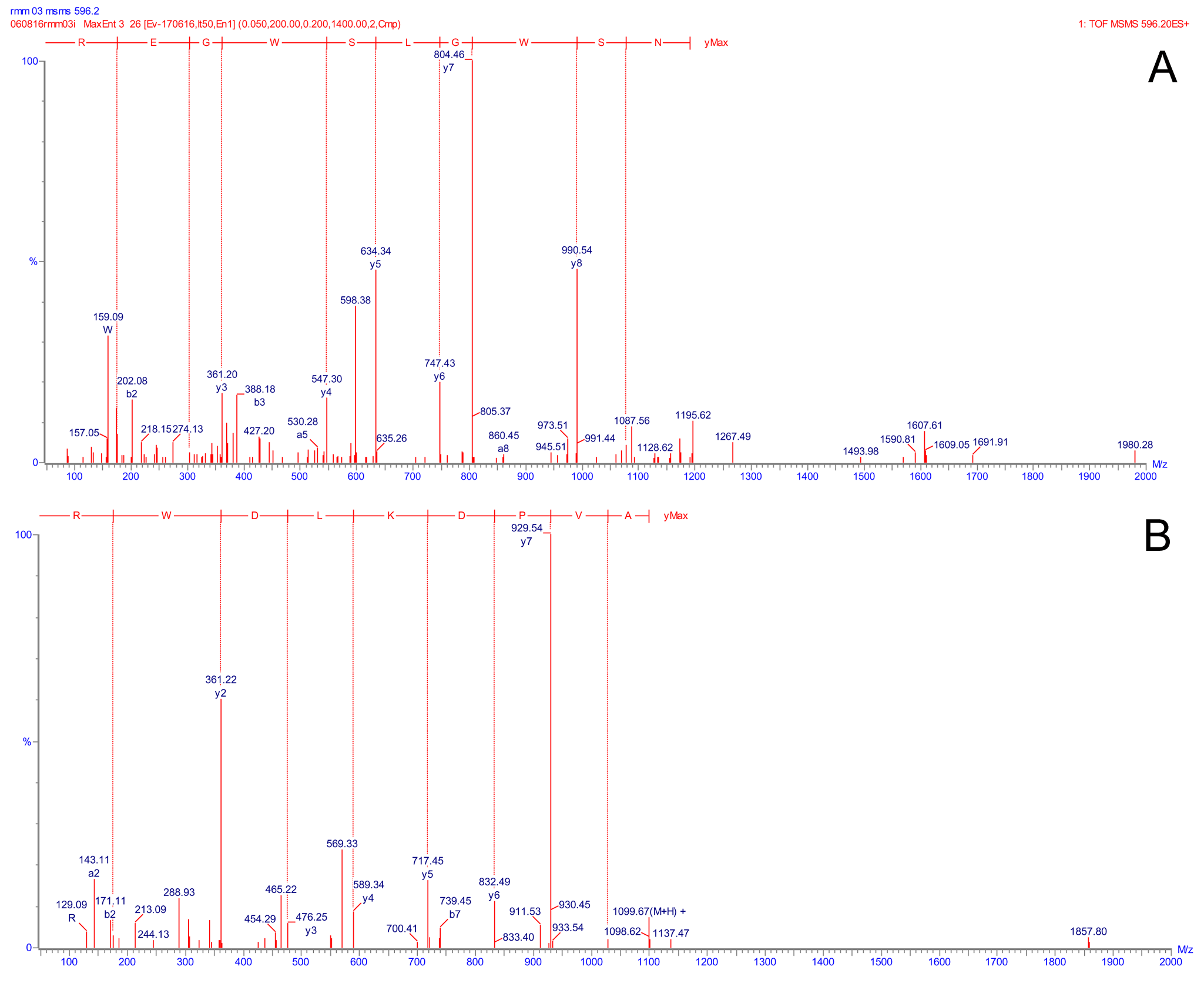

Supplement: Figure S9 — A) MSMS sequence analysis using peptide sequencer (MassLynx v. 5.0, Micromass, UK) from the fragmentation of a precursor ion m/z 596.27 (2+) representative of cathepsin L clade 1. Interpretation of the y and b ion series provided the peptide sequence NSWGLSWGER. B) MSMS sequence analysis using peptide sequencer (MassLynx v. 5.0, Micromass, UK) from the fragmentation of a precursor ion m/z 550.29 (2+) representative of cathepsin L clade 1. Interpretation of the y and b ion series provided the N-terminal peptide of the mature enzyme sequenced as AVPDKIDWR. (0.66 MB TIF) [file pntd.0000937.s009.tif]

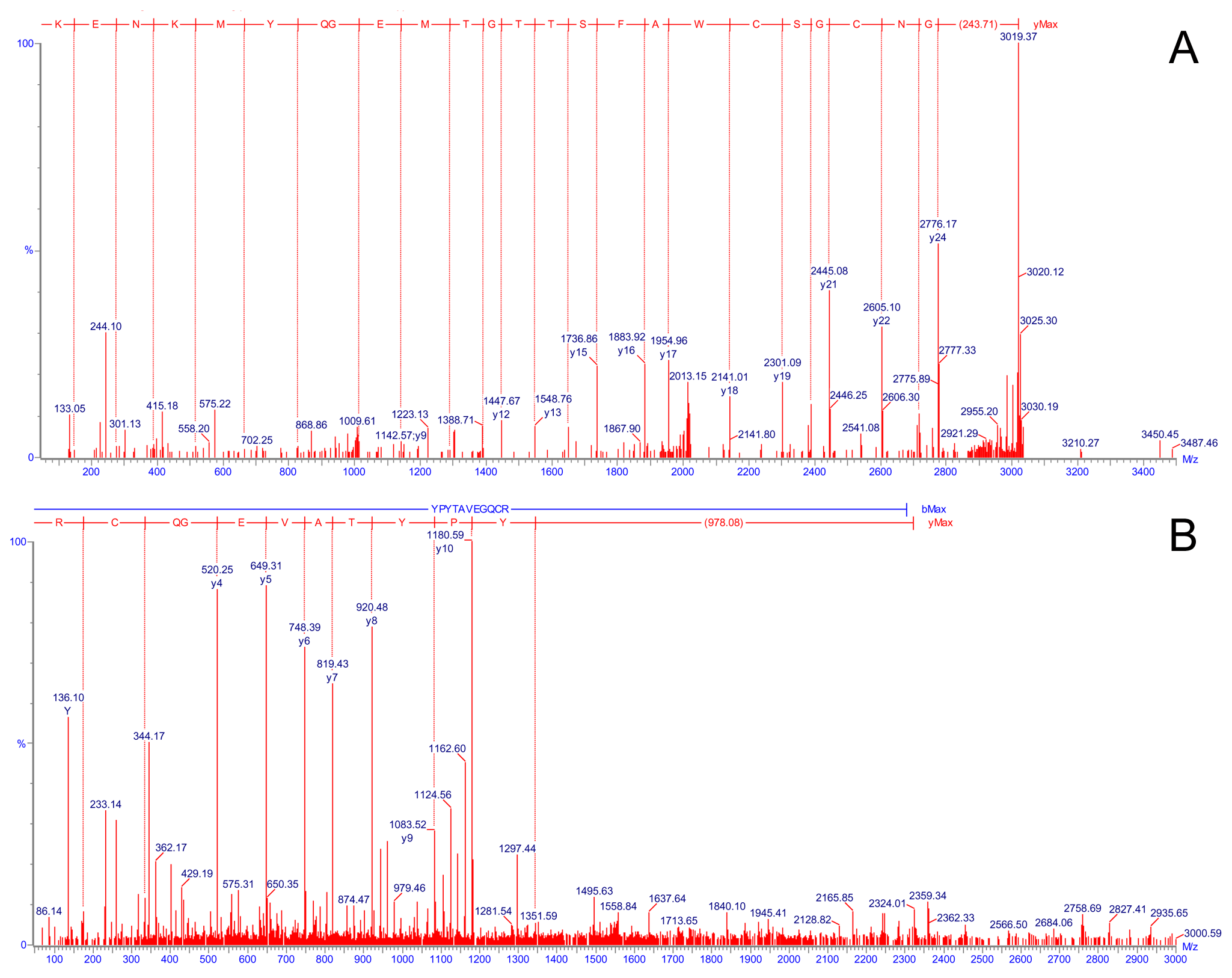

Supplement: Figure S10 — A) MSMS sequence analysis using peptide sequencer (MassLynx v. 5.0, Micromass, UK) from the fragmentation of a precursor ion m/z 1007.06 (3+) representative of cathepsin L clade 1B. Interpretation of the y and b ion series provided the peptide sequence GNCGSCWAFSTTGTMEGQYMKNEK. B) MSMS sequence analysis using peptide sequencer (MassLynx v. 5.0, Micromass, UK) from the fragmentation of a precursor ion m/z 774.68 (3+) providing the identification of spot 12. Interpretation of the y and b ion series provided the peptide sequence YPYTAVEGQCR. (0.95 MB TIF) [file pntd.0000937.s010.tif]

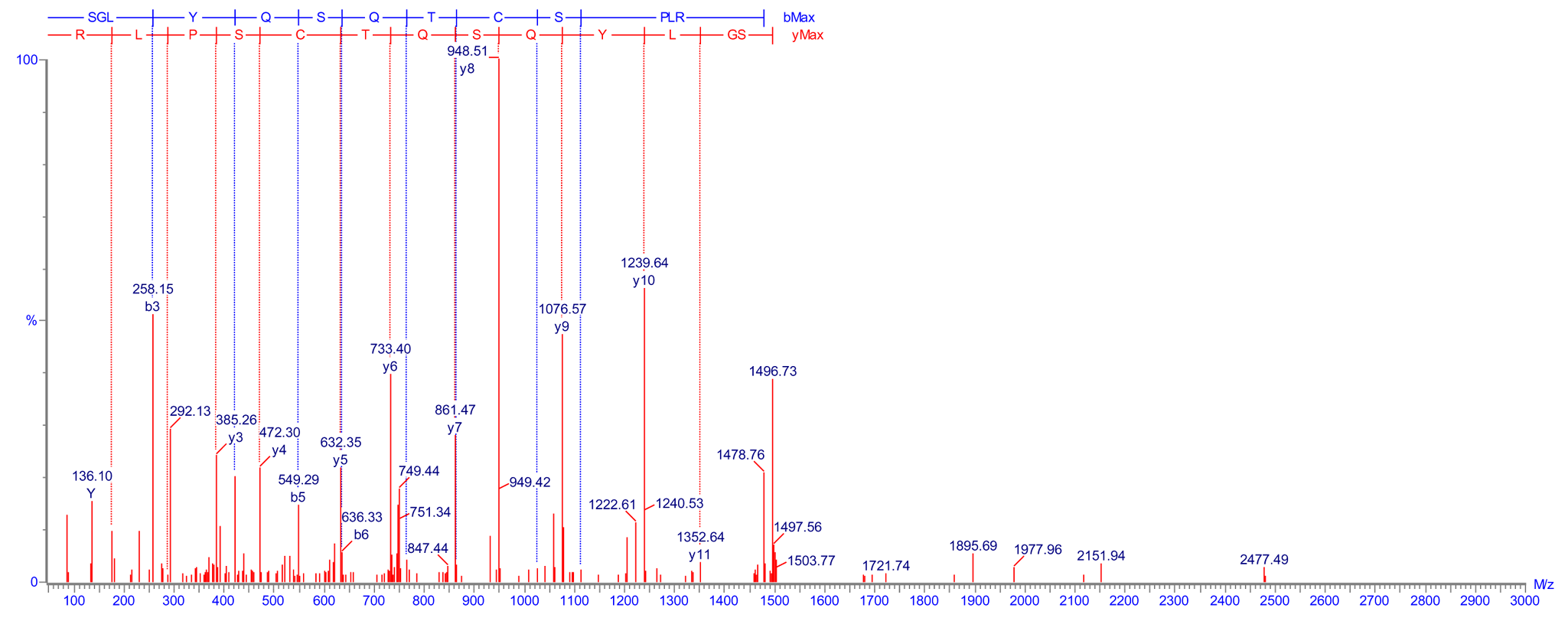

Supplement: Figure S11 — MSMS sequence analysis using peptide sequencer (MassLynx v. 5.0, Micromass, UK) from the fragmentation of a precursor ion m/z 748.86 (2+) providing the identification of spot 32. Interpretation of the y and b ion series provided the peptide sequence SGIYQSQTCSPLR. (0.43 MB TIF) [file pntd.0000937.s011.tif]
