# Supplementary material for: Towards Delineating Functions within the Fasciola Secreted Cathepsin L Protease Family by Integrating In Vivo Based Sub-Proteomics and Phylogenetics
Source: PLoS Negl Trop Dis. 2011 Jan 4;5(1):e937. doi: 10.1371/journal.pntd.0000937 (PMC3014944; doi:10.1371/journal.pntd.0000937)
Supplement: Table S1 — (0.01 MB PDF) [file pntd.0000937.s012.pdf]

Table S1. Cat L contribution to the ES products.

Clade		In Vitro (%)	In Vivo (%)
CL1		71.68	72.78
	A	42.21	35.98
	B	9.21	14.81
	C	0	0
	D	10.34	9.11
	NFD	9.92	12.88
CL2		20.60	15.07
CL3		0	0
CL4		0	0
CL5		5.52	5.61

Relative contribution of each cathepsin L clade and sub-clade to the expressed and secreted cathepsin L proteome identified *in vitro* and *in vivo*.
