# Supplementary material for: Towards Delineating Functions within the Fasciola Secreted Cathepsin L Protease Family by Integrating In Vivo Based Sub-Proteomics and Phylogenetics
Source: PLoS Negl Trop Dis. 2011 Jan 4;5(1):e937. doi: 10.1371/journal.pntd.0000937 (PMC3014944; doi:10.1371/journal.pntd.0000937)
Supplement: Table S2 — (0.05 MB PDF) [file pntd.0000937.s013.pdf]

Table S2. Peptide counts and protein coverage.

Spot Number	N^o Peptides	% Coverage
2	5	23
3	6	32
5	3	10
6	2	7
7	4	20
8	3	15
10	2 (1)	8 (3)
11	4	18
12	1	3
13	1	4
14	1	3
15	3	16
16	4	19
17	4	16
18A	4	19
18B	4	22
18C	3	16
19	4	18
20	4	15
21	1	4
22	3	16
23	4	18
24	2	8
25	1	4
27	3	12
28	3	13
29	1	5
30	3	14
31	2	9
32	1	4

Protein Identification data – the number of peptides (unique) assigned to each identification (N^o.) and the percentage coverage (%).
