# Supplementary material for: Towards Delineating Functions within the Fasciola Secreted Cathepsin L Protease Family by Integrating In Vivo Based Sub-Proteomics and Phylogenetics
Source: PLoS Negl Trop Dis. 2011 Jan 4;5(1):e937. doi: 10.1371/journal.pntd.0000937 (PMC3014944; doi:10.1371/journal.pntd.0000937)
Supplement: Table S3 — (0.13 MB PDF) [file pntd.0000937.s014.pdf]

Table S3. Identification of cathepsin L proteases from ES preparations by MSMS. Peptide sequences were used to search against Genbank (accession numbers provided) or a Translated EST database . Sequences with e values in red were not used to assign identity but provided confidence in the identification.

Spot Identifier	Precursor Mass	Precursor Charge	Peptide Mass	MS/MS Derived Peptides	NCBI BLAST e Value	Putitative Identity
2	863.81	2	1725.58	1-NSWGTWWGEAYTIR	2.80E-02	Cathepsin L-like
	883.36	2	1764.58	2-GNMCGIASLASVPMVAR	9.00E-08	
	604.20	3	1809.57	3-VTGYYTHSVTLEIELK	1.7	
	775.28	3	2323.77	4-YVGNYGCGGGYFEDAYEYLK	4.00E-07	
	798.37	3	2393.08	5-VLAVGYGSQDGTDYWIVK*	5.00E-09	
	939.36	3	2816.08	6-TESYYPYQAVEGP*	1.00E-04	
3	837.75	2	1673.78	1-QDGSGIASLASVPMVAR	3.00E-03	Secreted Cathepsin L2
	849.84	2	1697.58	2-ASASFDTQQLVDCTR	1.50E-02	
	863.75	2	1725.58	3-NSWGTWWGFPYGIR	0.95	
	1154.57	2	2306.78	4-YVGNYGCGGGYMENAYEYLK	8.00E-10	
	789.39	3	2392.18	5-LTHAVLAVGYGSQDGTDYWIVK	9.00E-13	
	1224.63	2	2446.98	6-NQGQCGSCADAFSTTGAVEGQFR	1.00E-08	
	945.00	2	2831.97	7-LGKDHTESYYPYQAVEGPCQYDGR	6.00E-11	
5	857.94	2	1713.78	1-ASASFSEQQLVD*	2.10E-02	Cathepsin L-like
	1117.04	2	2232.98	2-YMENAYEYLK*	2.10E-02	
	944.78	3	2832.28	3-QAVEGPCQYDGR*	2.00E-03	
6	724.85	2	1447.78	1-FGLETESSYPYR	2.00E-03	Cathepsin L-like/Cathepsin L
	586.32	3	1755.58	2-FSEQQLVDCSR*	5.90E-02	
	775.36	3	2322.88	3-MENAYEYLK*	0.36	
7	501.24	2	1000.38	1-VPESIDWR	4.6	Cathepsin L-like
	849.86	2	1697.58	2-ASASFSEQQLVDCTR*	1.00E-05	
	883.39	2	1764.58	3-GNMCGIASLASVQSGAAR	3.20E-02	
	604.27	3	1809.58	4-VTGYYTVHSGDEIELK*	5.00E-07	
	945.14	3	2831.98	5-GLQDHTESYYPYQAVEGQEQYDGR	1.90E-06	
	945.14	3	2831.98	6-PYQAVEGPCQYDGR*	2.80E-05	
8	724.80	2	1447.58	1-FGLETESSYPYR	2.00E-03	Cathepsin L-like/Cathepsin L
	878.83	2	1755.58	2-TSISFSEQQLVDCSR	7.00E-06	
	886.81	2	1771.78	3-TSISFSEQQLVGSMSR	9.00E-02	
	991.42	2	1980.78	4-DAPAFMASLASVPMVAQFP	1.00E-03	

Table S3. Continued.

Spot Identifier	Precursor Mass	Precursor Charge	Peptide Mass	MS/MS Derived Peptides	NCBI BLAST e Value	Putitative Identity
10	479.23	2	956.38	1-PDRIDWR	17	Cathepsin L-like/Cathepsin L and Cathepsin L/Cathepsin L-like
	724.81	3	1447.58	2-FGLETESSYPYR	2.00E-03	
	878.88	2	1755.58	3-TSISFSEQQLVDCSR*	8.00E-06	
	604.28	3	1809.58	4-VTGYYTVHLTATDELK	7.5	
	774.64	3	2320.78	5-YPYTAVEGQCR*	9.00E-03	
	829.66	3	2486.68	6-DFGNYGCNNLLFFLGWYGCR	7.5	
11	465.23	2	928.38	1-PDKIDWR	23	Cathepsin L and Cathepsin L/Cathepsin L-like
	550.29	2	1098.38	2-AVPDKIDWR	0.64	
	621.26	2	1240.38	3-NSWGSYWGER	7.90E-02	
	835.39	2	1668.58	4-GYYTVHSGSEVELK*	8.00E-05	
	874.41	2	1746.78	5-NMCGIASLA*	2.9	
	590.28	3	1767.58	6-TGYYTVHSGSEVELK*	1.00E-05	
	884.98	2	1767.78	7-VTGYYTVHSATTVELK	5.00E-03	
	890.43	2	1778.58	8-IASLASLPMVAR*	1.60E-03	
	1161.46	2	2320.78	9-QFGLETESSYPYTAVEGEGEE	7.00E-08	
	774.66	3	2320.78	10-YPYTAVEGQCR*	9.00E-03	
12	774.68	3	2320.78	1-YPYTAVEGQCR*	9.00E-03	Cathepsin L (Numerous types)
13	788.85	2	1575.58	1-QFGLETESSYPYR*	5.30E-04	Cathepsin L (Numerous types)
14	924.4	3	2770.18	1-QFGLETESSYP*	4.50E-02	Cathepsin L (Numerous types)
15	890.41	2	1778.78	1-GNFCGIASLASLPFVAR	6.00E-04	Cathepsin L
	1161.43	2	2320.78	2-QFGLETESSYPYTAVEGQGCR	8.00E-10	
	830.35	3	2487.88	3-ARVGSEGPAAVAVDVESPGCYNGAR	1.00E-04	
16	621.25	2	1240.38	1-NSWGSYWGER	0.28	Cathepsin L
	884.98	2	1767.78	2-VTGYYTVHSGSEVELK	1.00E-06	
	890.47	2	1778.78	3-GNFCGIASLASLPFVAR	6.00E-04	
	1161.49	2	2320.78	4-QFGLETESSYPYTAVEGQCR	4.00E-11	
	1016.39	3	3045.88	5-GSCWAFSTTG*	3.00E-01	
17	788.86	2	1575.58	1-QFGLETESSYPYR	5.30E-04	Cathepsin L/Cathepsin L-like/Cathepsin/Cathepsin L1
	884.94	2	1767.78	2-VTGYYTVHSGSEVELK	1.00E-06	
	890.39	2	1778.78	3-GNFCGIASLASLPFVAR	6.00E-04	
	1161.54	2	2320.98	4-ETESSYPYTAVEGQCR*	3.00E-07	

Table S3. Continued.

Spot Identifier	Precursor Mass	Precursor Charge	Peptide Mass	MS/MS Derived Peptides	NCBI BLAST e Value	Putitative Identity
18A	465.28	2	928.34	1-PDKIDWR	23	Cathepsin L1
	550.27	2	1098.58	2-AVPDKIDWR	0.64	
	596.27	2	1190.38	3-NSWGLSWGER	6.80E-02	
	580.91	3	1739.68	4-VTGYYTVHSGSEAELK	2.00E-05	
	590.36	3	1767.87	5-VTGYYTVHSGSEVELK	1.00E-06	
	653.66	3	1957.78	6-LSAPWCIASLASLPMVAR	1.80E-02	
	765.37	3	2292.88	7-ASLPMVARFP*	0.35	
	1007.06	3	3018.88	8-GNCGSCWAFSTTGTMEGQYMKNEK*	6.00E-16	
18B	465.24	2	928.38	1-PDKIDWR*	23	Cathepsin L
	550.29	2	1098.58	2-AVPDKIDWR*	0.64	
	590.36	3	1767.87	3-VTGYYTVHSGSVELK	1.00E-06	
	774.62	3	2320.78	5-QFGLETESSYPYTAVEGQCR	4.00E-11	
	825.05	3	2471.98	6-GPAAVAVDVESDF*	7.00E-03	
	1010.65	4	4039.57	7-NCGSCWAFSTTGTMEGQYMKNER*	3.00E-15	
18C	890.43	2	1778.78	1-GNMCGIASLASLSAQGAR	1.10E-02	Cathepsin L (Numerous types)
	1153.02	2	2303.98	2-ETESSYPYTAVEGQCR*	3.00E-06	
	1010.98	4	4039.55	3-NCGSCWAFSTTGTMEGQYMKNER*	3.00E-15	
19	596.27	2	1190.38	1-NSWGLSWGER	6.90E-02	Cathepsin L
	621.27	2	1240.38	2-NSWGSYWGER	2.80E-02	
	590.29	3	1767.58	3-TGYYTVHSGSEVELK*	1.00E-05	
	884.87	2	1767.78	4-VTGYYTVHSTATVELK	1.50E-02	
	890.40	2	1778.78	5-GNMCGIASLASLPMVAR	9.00E-08	
	774.67	3	2320.77	6-QFGLETESSYPYTAVLCAQTN	5.00E-06	
20	788.86	2	1575.58	1-QFGLETESSYPY*R*	5.30E-04	Cathepsin L/Cathepsin L-like/Cathepsin/Cathepsin L1
	590.28	3	1767.58	2-TGYYTVHSGSEVEL*	8.00E-05	
	890.41	2	1778.78	3-GNMCGIASLASLKTGGAR	3.20E-02	
	774.66	3	2320.78	4-GNRTFTQSSYPYTAVEGTELT	4.90E-02	
21	910.05	3	2726.98	1-FGLETESSYPYT*	3.00E-03	Cathepsin L (Numerous Types)
22	755.35	2	1508.58	1-GYYTLHSGNEAGLK	2.90E-04	Cathepsin L-like
	788.84	2	1575.58	2-QFGLETESSYPY*R**	5.30E-04	
	805.86	2	1609.58	3-TGYYTLHSGNEAGLK	3.70E-05	
	570.59	2	1708.48	4-VTGYYTLHSGNEAGLK*	4.80E-06	
	882.41	2	1762.78	5-IASLASLPMVAR*	1.60E-03	
	890.40	2	1778.78	6-GNMCGIASLASL*	1.20E-02	

Table S3. Continued.

Spot Identifier	Precursor Mass	Precursor Charge	Peptide Mass	MS/MS Derived Peptides	NCBI BLAST e Value	Putitative Identity
23	465.28	2	928.34	1-PDKIDWR	23	Cathepsin L/Cysteine Protease
	550.27	2	1098.58	2-AVPDKIDWR	0.64	
	621.31	2	1240.38	3-NSWGSYWGER	0.028	
	890.42	2	1778.78	4-MCGIASLASLPMVAR*	6.00E-06	
	1152.98	2	2303.78	5-ETESSYPYTAVEGQCR*	3.00E-07	
	1161.64	2	2321.98	6-QFGLETESSYGYSQPEGQCR	2.00E-05	
	783.38	3	2346.88	7-GYGTQGGTDYWIVK*	2.00E-05	
	825.03	3	2471.98	8-LNVKEGAPAVAVDVESDEAAFGER	0.68	
24	788.85	2	1575.58	1-QFGLETESSYPYR*	5.30E-04	Cathepsin L (Numerous types)
	890.41	2	1778.78	2-GFCNGIASLASLPMVAR	7.40E-03	
25	788.85	2	1575.58	1-QFGLETESSYPYR*	5.30E-04	Cathepsin L (Numerous types)
	689.32	3	2064.88	2-ASLASLPMVAR*	0.19	
27	755.32	2	1508.58	1-GYYTLHSGNEAGLK	2.90E-04	Cathepsin L-like
	788.89	2	1575.58	2-QFGLEGVGLQLPYR	2.00E-03	
	570.57	3	1708.78	3-VTGYYTLHSDANAGLK	4.30E-02	
	890.42	2	1778.78	4-NMCGIASLASLPMVAR*	1.50E-06	
	684.29	3	2048.98	5-GDKSGIASLASLPFVAR	9.00E-02	
28	465.20	2	928.38	1-PDKIDWR	23	Cathepsin (Precursor)
	748.82	2	1495.58	2-SGIYQSQTCSPLR	5.00E-04	
	844.84	2	168.583	3-GNESGIASLASLPFVAR	6.70E-02	
	590.27	3	1767.58	4-VTGYYTVHCVEAELK	7.00E-01	
	774.63	3	2320.78	4-QFGLETESSYPYTAVEGGASTQ	9.00E-08	
29	590.28	3	1767.78	1-VTGYYTVHSGSEVELK	1.00E-06	Cathepsin L/Cathepsin L-like
30	755.30	2	1508.58	1-GYYTLHSGNEAGLK	2.90E-04	Cathepsin L-like
	788.84	2	1575.58	2-QFGLETESSYPYR	5.30E-04	
	805.81	2	1609.58	3-TGYYTLHSGNEAGLK	3.70E-05	
	570.59	3	1708.48	4-VTGYYTLHSGNEAGLK	4.80E-06	
	874.30	2	1746.58	5-GNMCGIASLASLPMVAR	9.00E-08	

Table S3. Continued.

Spot Identifier	Precursor Mass	Precursor Charge	Peptide Mass	MS/MS Derived Peptides	NCBI BLAST e Value	Putitative Identity
31	465.20	2	928.38	1-**PDKIDWR**	23	Cathepsin L-like
	550.20	2	1098.38	2-**AVPDKIDWR**	0.64	
	755.31	2	1508.58	3-**GYYTLHSGNEAGLK**	2.90E-04	
	789.84	2	1575.58	4-**QFGLETESSYPY*R***	5.30E-04	
	805.82	2	1609.58	5-**TGYYTLHSGNEAGLK**	3.70E-05	
	570.61	3	1708.78	6-**VTGYYTLHSGNEAGLK**	4.80E-06	
	855.41	2	1708.78	7-**VTGYYTLHSGNEAGLK**	4.80E-06	
32	748.86	2	1495.58	1-**SGIYQSQTCSPLR**	5.00E-04	Secreted Cathepsin L1/Cathepsin L/Cathepsin (Precursor)
